# Wharton's Jelly-Derived Mesenchymal Stromal Cells and Fibroblast-Derived Extracellular Matrix Synergistically Activate Apoptosis in a p21-Dependent Mechanism in WHCO1 and MDA MB 231 Cancer Cells* In Vitro*

**DOI:** 10.1155/2016/4842134

**Published:** 2016-01-10

**Authors:** Kevin Dzobo, Matjaz Vogelsang, Nicholas E. Thomford, Collet Dandara, Karlien Kallmeyer, Michael S. Pepper, M. Iqbal Parker

**Affiliations:** ^1^International Centre for Genetic Engineering and Biotechnology (ICGEB), Cape Town Component, Wernher and Beit Building (South), UCT Campus, Anzio Road, Observatory, Cape Town 7925, South Africa; ^2^Division of Medical Biochemistry and Institute of Infectious Disease and Molecular Medicine, Faculty of Health Sciences, University of Cape Town, Anzio Road, Observatory, Cape Town 7925, South Africa; ^3^Perlmutter Cancer Centre, New York University, School of Medicine, New York, NY 10016, USA; ^4^Division of Human Genetics, Faculty of Health Sciences, University of Cape Town, Anzio Road, Observatory, Cape Town 7925, South Africa; ^5^Department of Immunology and Institute for Cellular and Molecular Medicine, Faculty of Health Sciences, University of Pretoria, Gezina, Pretoria 0002, South Africa

## Abstract

The tumour microenvironment plays a crucial role in tumour progression and comprises tumour stroma which is made up of different cell types and the extracellular matrix (ECM). Mesenchymal stromal cells (MSCs) are part of the tumour stroma and may have conflicting effects on tumour growth. In this study we investigated the effect of Wharton's Jelly-derived MSCs (WJ-MSCs) and a fibroblast-derived ECM (fd-ECM) on esophageal (WHCO1) and breast (MDA MB 231) cancer cells* in vitro*. Both WJ-MSCs and the fd-ECM, alone or in combination, downregulate PCNA, cyclin D1, Bcl-2, Bcl-xL, and MMPs and upregulate p53 and p21. p21 induction resulted in G2 phase cell cycle arrest and induced apoptosis* in vitro*. Our data suggest that p21 induction is via p53-dependent and p53-independent mechanisms in WHCO1 and MDA MB 231 cells, respectively. Vascular endothelial growth factor, Akt, and Nodal pathways were downregulated in cancer cells cocultured with WJ-MSCs. We also demonstrate that WJ-MSCs effects on cancer cells appear to be short-lived whilst the fd-ECM effect is long-lived. This study shows the influence of tumour microenvironment on cancer cell behaviour and provides alternative therapeutic targets for potential regulation of tumour cells.

## 1. Introduction

Mesenchymal stromal cells (MSCs) are a heterogeneous population of stromal derived cells that contain a subpopulation of multipotent progenitor cells defined according to their ability to self-renew and differentiate into tissues of mesodermal and nonmesodermal lineages [1–3]. MSCs have the ability to influence the growth of other cells through the release of growth factors, cytokines, and antifibrotic mediators. Initially isolated from the bone marrow, MSCs are now obtained from different sources such as the bone marrow, adipose tissue, and the umbilical cord [4–7]. MSCs have the ability to repair and regenerate various types of damaged tissues, hence their use in cell therapy and tissue regeneration [8, 9]. In addition, MSCs have received attention for their role in tumour growth and metastasis [10–13]. Several studies have shown contradictory results regarding the role of MSCs during tumour growth and metastasis [14–16]. This is mainly due to the involvement of many factors in determining whether MSCs promote or inhibit tumour growth. These factors include the type of tumour being investigated, the different methodologies used in the experiments, method of MSCs isolation, the number and origin of MSCs used, and whether the study is performed* in vitro* or* in vivo* [17].

Several studies have shown that MSCs have tumour growth-promoting functions in the tumour microenvironment through secretion of growth factors, induction of angiogenesis, and the creation of cancer stem cell niches [18, 19]. However, other studies have shown that MSCs can exert an antitumorigenic effect in Kaposi sarcoma and hepatoma animal models which has led to their use in the treatment of autoimmune diseases and chronic inflammation [20–22]. MSCs also inhibit primary tumour growth and metastasis formation in mice resulting in prolonged survival [14, 15, 23]. Implantation of human skin-derived stem cells into glioblastoma has been observed to result in reduced tumour vessel density and angiogenic sprouts [24]. MSCs elicit antimetastatic effects by acting on the vascular endothelial growth factor, Wnt, Nodal, and PI3-K signaling pathways and through secretion of cytokines such as IL2 and IL15 [25].

The stromal microenvironment plays an active role during cancer development and metastasis [17, 26, 27]. During tumour progression an environment that was inhibitory to tumour growth may become permissive and start to actively promote tumour cell growth and allowing tumours to lose contact inhibition and become invasive [17, 27]. As tumour cells gain greater access to the mesenchymal stroma they come into contact with many cell types including the MSCs and an extracellular matrix (ECM) synthesised by stromal cells such as fibroblasts. Fibroblasts and MSCs are known to be recruited to or invade the tumour stroma and to change the tumour ECM [16, 27, 28]. It is therefore important to use stromal or mesenchymal 3D ECMs to represent the* in vivo* tumour microenvironment as cancer cells are likely to come into contact with these ECMs during the early stages of cancer growth, invasion, and metastasis. In addition, many studies have shown that* in vitro* 2D models do not properly predict cancer cell drug responses and chemoresistance [26, 29–31].

One of the promising reprogramming models investigated in our laboratory is the role of the fibroblast microenvironment [32–34]. Little is known about the direct effects that a fibroblast-derived ECM has on esophageal and breast cancer cells. We believe that the fd-ECM can be used as 3D substrate to study tumour-associated ECM-induced responses such as cellular growth and invasion. To date no study has investigated the effects of WJ-MSCs and the fd-ECM, alone or in combination, on oesophageal and breast cancer cells in terms of expression profiles of genes associated with proliferation, cell cycle progression, apoptosis, and tumorigenic signaling. The aim of this study was therefore to simultaneously investigate the influences imparted by Wharton's Jelly-derived MSCs (WJ-MSCs) and the fd-ECM on genes that are highly expressed in WHCO1 and MDA-MB 231 cancer cells and these include PCNA, Bcl-2, Bcl-xL, p53, p21, proteases, and their inhibitors [10, 26, 27, 29, 30]. We show in this study that both WJ-MSCs and the fd-ECM inhibit the proliferation of WHCO1 and MDA MB 231 cancer cells and induced G2 phase cell cycle arrest leading to apoptosis. The effects of WJ-MSCs on cancer cells appear to be short-lived whereas the fd-ECM effects appear to be long-lived. The fibroblastic microenvironment together with MSCs provides an alternative therapeutic entity for the potential regulation of aggressive tumour cells.

## 2. Materials and Methods

### 2.1. Reagents

Cell culture reagents were obtained from GIBCO BRL Life Technologies (Gaithersburg, MD, USA). Transfection and the SDS-PAGE molecular weight standards were from BioRad (California, USA). Ammonium hydroxide was purchased from Merck Biosciences (Darmstadt, Germany). Other chemicals and solvents if not otherwise specified were purchased from either Sigma-Aldrich (St Louis, MO, USA) or Merck Biosciences (Darmstadt, Germany).

### 2.2. Isolation and Culture of Wharton's Jelly-Derived MSCs

Human umbilical cords were collected from full-term births after either caesarean section or normal vaginal delivery with informed consent using the guidelines approved by Institutional Ethics Committee at the University of Pretoria, South Africa. Mesenchymal stromal cells from Wharton's Jelly (WJ) of the umbilical cord were isolated as previously described [35, 36]. Briefly, umbilical cords were cut into 1 to 2 mm^3^ pieces, enzymatically digested for 1 hr at 37°C with 3 mg/mL of collagenase type I (Sigma-Aldrich). After the enzymatic treatment, cells were filtered through a cell strainer and centrifuged at 1500 rpm for 5 mins. The pellets were collected as WJ-MSCs and plated in 100 mm tissue culture dishes in KnockOut DMEM (GIBCO) supplemented with 10% FBS, fibroblast growth factor 100 U/mL penicillin, and 100 *μ*g/mL streptomycin. For the experiments described in this paper, 4 WJ-MSCs preparations were used separately or were cultured separately and then pooled at passages 5-6. Pooled MSCs were expanded in similar culture conditions as described before. WJ-MSCs were analyzed for MSC markers before and after pooling using flow cytometry. Individual donor MSCs and pooled MSCs exhibited no significant differences in growth properties, phenotypes, and gene expression patterns (data not shown). We also compared individual donor MSCs and pooled MSCs (same passage) for their effect on both WHCO1 and MDA MB 231 cell proliferation and gene expression and we observed no significant differences (data not shown).

### 2.3. Cell Culture and Preparation of hMSC-Conditioned Medium

All the cells were maintained under standard conditions. Human WJ-derived MSCs from healthy adults were cultured in *α*-minimal essential medium (*α*-MEM) (GIBCO, Grand Island, NY) supplemented with 10% heat-inactivated fetal bovine serum (FBS) (GIBCO) and 100 U/mL penicillin and 100 mg/mL streptomycin (GIBCO). For preparation of hMSC-conditioned medium (hMSC-CM), MSCs were seeded on 100 mm culture dishes (5 × 10^5^ cells) and grown to 80% confluence and fresh medium was added. After 3 days of incubation the medium was collected, filtered through a 0.22 *μ*m filter, and stored at −80°C until used as the human MSC conditioned medium (hMSC-CM). Human lung fibroblast WI38 cells (ATCC, USA), human breast cancer cell line, MDA-MB-231 (KCLB), and oesophageal cancer cell line, WHCO1, were cultured in Dulbecco's Modified Eagle's Medium (DMEM; GIBCO) supplemented with 10% heat-inactivated FBS, 100 U/mL penicillin, and 100 mg/mL streptomycin. The oesophageal cancer cell-line WHCO1 was derived from a biopsy of primary oesophageal squamous cell carcinoma of South African origin [37]. MSCs were routinely characterised by flow cytometry for a variety of intracellular markers and cell surface antigens. Mesenchymal stromal cell isolates that exhibited positive expression of CD105, CD90, CD73, and CD44 and negative expression for CD45, CD14, and CD34 were used in all experiments. Cells were used for experiments between passages 4 and 10.

### 2.4. Preparation of fd-ECM Coated Dishes

The fd-ECM was prepared from normal WI38 fibroblasts as previously described [26, 31–34]. Fibroblasts are used for the preparation of the ECM as they are known to be recruited to tumour sites and do contribute, negatively or positively, to tumour growth [26, 27]. Briefly, WI38 fibroblasts were cultured in DMEM supplemented with 10% heat-inactivated fetal bovine serum, 2 mM L-glutamine, 100 U/mL penicillin, and 100 *μ*g/mL streptomycin. Ascorbic acid (50 *μ*g/mL) was added every alternate day. Cells were grown to 8 days after confluence after which they were washed with PBS and incubated with 20 mM ammonium hydroxide containing 0.005% Triton X-100 (Sigma) for 1 minute at room temperature to remove cells. The resulting fd-ECM was treated with DNase I (100 U/mL; Sigma) for 1 hour at 37°C. The fd-ECM was washed with sterile PBS three times and stored in 2 mL of PBS containing 100 U/mL penicillin and 100 *μ*g/mL streptomycin (Invitrogen, USA) at 4°C. The fd-ECM was used immediately or within two weeks of preparation.

### 2.5. Coculture Assay

Cells were cocultured in 6-transwell plates (0.4 *μ*m pore, polycarbonate membrane, Costar, Corning, Cambridge, USA). hMSCs (5 × 10^5^ cells) were loaded in the upper insert and WHCO1 and MDA MB 231 cancer cells (5 × 10^5^ cells) were plated into the lower compartment of the 6-well plates with or without fd-ECM. The control group had empty inserts (no cells) containing a mixture of hMSCs medium and cancer cell medium (1 : 1). When hMSC-CM was used, WHCO1 and MDA MB 231 cells were plated in 6-well plates and a mixture containing hMSC-CM and cancer cell medium (1 : 1) was added. For longer incubation periods, medium was changed every 3 days. After the indicated culture period, both cancer cells and hMSCs were collected, counted, and used in RT PCR, Western blot, and flow cytometric analysis in triplicate. To study the specificity of the effect of WJ-MSCs on cancer cells, WHCO1 and MDA MB 231 cells were cocultured with WHCO1 and MDA MB 231 cells (insert), respectively. Coculture was also done with WI38 fibroblasts (insert).

### 2.6. Western Blot Analysis

Cell layers were washed with cold PBS and lysed in RIPA buffer (10 mM Tris-HCl pH 7.6, 10 mM NaCl, 3 mM MgCl_2_, and 1% (v/v) Nonidet P-40). Total protein concentration was determined using the BCA assay (Thermo Scientific, Illinois). Total cell lysates (50 *μ*g) were separated by electrophoresis on 10% polyacrylamide/SDS gels under reducing conditions (50 mM *β*-mercaptoethanol). After electrophoresis, proteins were transferred to nitrocellulose membranes and blocked in 5% fat-free milk in Tris Buffered Saline (TBS) containing Tween-20 for 1 hour. The membranes were incubated overnight at 4°C with the following primary antibodies: anti-Ki67 antibody, anti-p53 antibody, anti-p21 antibody, anti-p-ERK 1,2 antibody, anti-ERK 2 antibody, anti-p-Smad2 antibody, anti-Smad2 antibody, anti-Nodal antibody, anti-type I collagen antibody, anti-MMP2 antibody, anti-MMP9 antibody, anti-cyclin D1 antibody, anti-Bcl-xL antibody, anti-Bcl-2 antibody, anti-PCNA antibody, anti-*α*-smooth muscle actin antibody, anti-*β*-catenin antibody, anti-NFk-*β* antibody, anti-TGF-*β* antibody, anti-p-Akt antibody, anti-Akt antibody, anti-p-Raf1 antibody, anti-Raf1 antibody, anti-cleaved PARP, anti-cleaved caspases 3 and 9, and anti-GAPDH antibody (Santa Cruz Biotechnology, California, USA). After several washes in TBS-Tween buffer, the membranes were incubated with the specific horseradish peroxidase-conjugated (HRP) secondary antibodies (Bio-Rad) and detected using LumiGLO substrate (KPL, Gaithersburg, USA). All experiments were done in triplicate and repeated three times.

### 2.7. RNA Preparation and RT-qPCR

Total RNA was extracted from cells according to the procedure of Chomczynski and Sacchi [38] using Trizol reagent (BioRad, California). Complementary DNA (cDNA) was generated using ImProm II reverse transcriptase (Promega, Madison) according to the manufacturers' instructions. Quantitative PCR reactions were performed and monitored using the LightCycler 480 II (Roche). cDNA samples (2.0 *μ*L in a total volume of 20 *μ*L) from triplicate samples were analyzed using the primers listed in Supplemental Table S1 (in Supplementary Material available online at http://dx.doi.org/10.1155/2016/4842134). Thermocycling for all targets was carried out under the following conditions: initial denaturation at 94°C for 10 minutes followed by 35 cycles of 94°C for 20 s, 55°C for 15 s, and 72°C for 20 s. Relative gene expression was computed for each sample by comparison to the respective controls. RT-qPCR was done in triplicate and repeated three times.

### 2.8. Flow Cytometry/Cell Cycle Analysis

Control and treated WHCO1, MDA MB 231, and WJ-MSC cells were detached and processed for flow cytometry analysis. Briefly, cells were washed twice with cold PBS and fixed in ice-cold 70% ethanol for 60 minutes at 4°C. The cells were then washed twice with PBS, treated with RNase A (10 *μ*g/mL) for 30 minutes at 37°C, and stained with 200 *μ*L of propidium iodide (PI) stock solution (50 *μ*g/mL propidium iodide, 3.8 mM sodium triphosphate in PBS). Flow cytometric analysis was done with a FACScan cell sorter (Becton Dickinson, Franklin Lakes, NJ, USA). Ten thousand cells were collected and cell cycle profiles were calculated by using the CellQuest and ModFit software (Becton Dickinson, NJ, USA).

### 2.9. Immunophenotyping

Wharton's Jelly-derived MSCs, treated as described above, were subjected to flow cytometric analysis at passages 1–10. Antibodies to CD44-FITC, CD45-FITC, CD73-FITC, CD90-FITC, and CD105-FITC (Abcam, USA) were used to mark cell surface epitopes. All analyses were standardised against negative control cells incubated with isotype-specific IgG1-FITC (Santa Cruz). The number of cells staining positive for a marker was determined by the percentage of cells present within a gate established using the FITC-conjugated isotype-matched control. At least 50 000 events were acquired on a FACScan cell sorter (Becton Dickinson, NJ, USA). For osteogenic and chondrogenic differentiation, MSCs were incubated with the respective media (Life Technologies) for up to 21 days. The medium was replaced twice every week. For osteogenic differentiation mineralization was visible as red stained calcium deposition. For chondrogenic differentiation, proteoglycans were stained with toluidine blue O which was visible as purple. For adipogenic differentiation, Oil Red O staining was done and lipid droplets were quantified.

### 2.10. siRNA-Mediated Gene Silencing Assay

Small interfering RNAs (siRNAs) against p21 and p53 were purchased from Santa Cruz Biotechnology (California, USA). The siRNA Transfection Reagent Transfection (Biorad) was used to transfect the siRNA into cells at a final concentration of 100 nM. Transfection complexes were prepared in media and added simultaneously dropwise to all wells. After 6 hours the medium was changed to fresh conditioned media or culture media. To maintain and increase knockdown efficiency subsequent transfections were carried out every 2 days for longer incubation periods. The cells were harvested and Western blot analysis was performed to quantify proteins levels.

### 2.11. Gelatin Zymography

Zymographic analysis of protease activity was performed as described before [33, 39]. Polyacrylamide gels contained 1 mg/mL gelatin. Media samples, from control, coculture, and fd-ECM experiments, were added to an equal volume of 2x loading buffer (nonreducing) and electrophoresis was done at 25 mA for 2 hrs. After electrophoresis gels were washed twice, for 30 minutes each, in 200 mL of 2.5% Triton X-100 with constant stirring. The gels were incubated in incubation buffer (50 mM Tris-HCl, pH 7.5, 5 mM CaCl_2_, and 0.2 M NaCL) for 18 hrs at 37°C. Gels were stained with Coomassie brilliant blue RR-250 in 20% methanol and destained in 15% acetic acid and 10% methanol. Gelatinolytic activity was evident as transparent zones in the blue gels. The area of the cleared zones was analyzed with the Fluor-S Multi-imager (Bio-Rad).

### 2.12. Immunofluorescence

Immunofluorescence microscopy analysis was done on cells grown in 6-well plates with or without the fd-ECM or coculture. Culture media were removed; cells were washed twice with PBS, fixed with 4% paraformaldehyde for 15 mins, and permeabilized using triton X-100 in sodium citrate. Blocking was done using 5% milk in Tris buffered saline containing Tween 20 for 1 hour and then incubation with anti-Bcl-2 antibody or isotopic control antibody. Washes were done using PBS, and the secondary antibody was tagged with FITC. DAPI staining was done as well. Slides were viewed under a Carl Zeiss microscope.

### 2.13. Statistical Analysis

Statistical analysis was performed using GraphPad Prism version 5. Data were expressed as means ± SD. The paired Student's *t*-test was used to evaluate statistical differences between control cells and treated cells. Values of *P* < 0.05 were considered significant. All experiments were repeated at least three times.

## 3. Results

### 3.1. Effect of WJ-MSCs and fd-ECM on Cancer Cell Gene Expression

**Figure 1:**
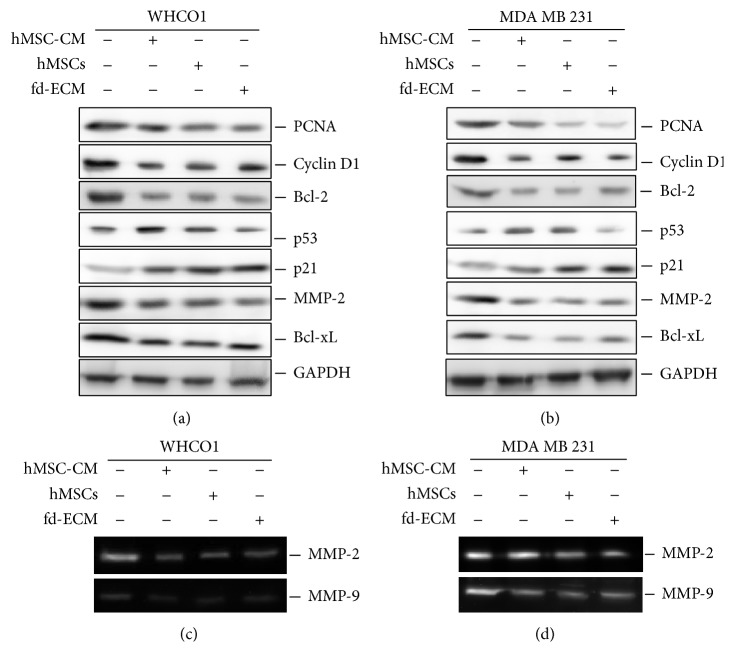
MSCs and fd-ECM inhibit cellular gene expression in WHCO1 and MDA MD 231 cells. (a) Western blot analysis of lysates from WHCO1 cells cocultured with MSCs or cultured on an fd-ECM for 24 hrs showing expression of PCNA, cyclin D1, Bcl-2, p53, p21, MMP-2, MMP-9, and Bcl-xL. (b) Western blot analysis of lysates from MDA MB 231 cells cocultured with MSCs or cultured on an fd-ECM for 24 hrs showing expression of the same genes as in (a). Glyceraldehyde 3-phosphate dehydrogenase (GAPDH) was used as a loading control. ((c)-(d)) MMP-2 and MMP-9 gelatinolytic activities in WHCO1 (c) and MDA MB 231 (d) in media samples after 24 hrs of incubation at 37°C as described in Section 2. Gelatinolytic activity was observed as clear bands in the gels. Experiments were performed in triplicate and repeated at least 2 times.

**Figure 2:**
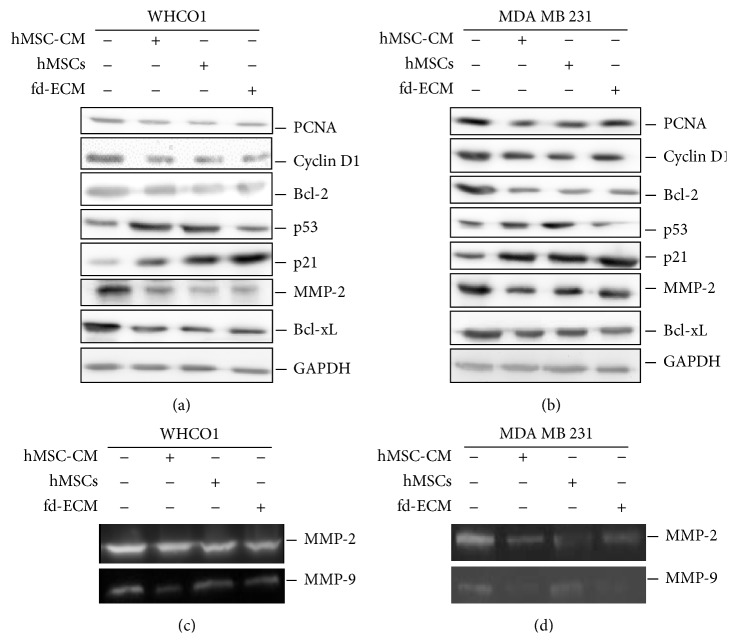
MSCs and fd-ECM inhibit cellular gene expression in WHCO1 and MDA MD 231 cells. (a) Western blot analysis of lysates from WHCO1 cells cocultured with MSCs or cultured on an fd-ECM for 48 hrs showing expression of PCNA, cyclin D1, Bcl-2, p53, p21, MMP-2, MMP-9, and Bcl-xL. (b) Western blot analysis of lysates from MDA MB 231 cells cocultured with MSCs or cultured on an fd-ECM for 48 hrs showing expression of the same genes as in (a). Glyceraldehyde 3-phosphate dehydrogenase (GAPDH) was used as a loading control. ((c)-(d)) MMP-2 and MMP-9 gelatinolytic activities in WHCO1 (c) and MDA MB 231 (d) in media samples after 48 hrs of incubation at 37°C as described in Section 2. Gelatinolytic activity was observed as clear bands in the gels. Experiments were performed in triplicate and repeated 3 times.

**Figure 3:**
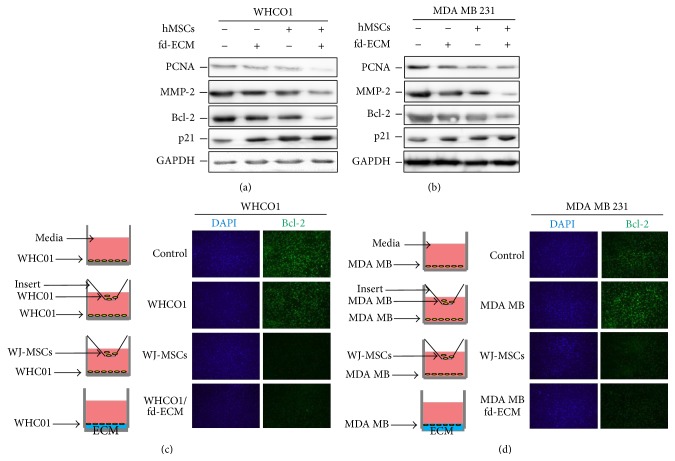
MSCs and the fd-ECM synergistically downregulate WHCO1 and MDA MB 231 gene expression. (a) Western blot analysis of lysates from WHCO1 cells cocultured with MSCs, cultured on an fd-ECM, or in combination, for 48 hrs showing expression of PCNA, Bcl-2, MMP-2, and p21. (b) Western blot analysis of lysates from MDA MB 231 cells cocultured with MSCs, cultured on an fd-ECM, or in combination, for 48 hrs showing expression of the same genes as in (a). (c) Immunofluorescence staining for Bcl-2 in WHCO1 cells after coculture with WHCO1 cells (second panel) and WJ-MSCs (third panel) and also cultured on fd-ECM (fourth panel). (d) Immunofluorescence staining for Bcl-2 in MDA MB 231 cells after coculture with MDA MB 231 cells (second panel) and WJ-MSCs (third panel) and also cultured on fd-ECM (fourth panel).

We have previously shown that the fd-ECM directs embryonic stem cell differentiation via the endodermal lineage [34] and we therefore explored the possibility that this* in vitro* 3D ECM synthesised by fibroblasts, alone or in combination with WJ-MSCs, could reprogram the tumour cell phenotype. WJ-MSCs were characterised by identification of cell surface markers by flow cytometry using FITC labeled antibodies. WJ-MSCs were positive for CD44, CD73, CD90, and CD 105 whilst they were negative for CD45 (Supplemental Figure S1 A–F). WJ-MSCs were able to differentiate into osteogenic and chondrogenic lineages after cultivation in respective media (Life Technologies) (Supplemental Figure S2). MDA MB 231 and WHCO1 cancer cells were treated with MSC-CM, cocultured with MSCs, and plated on fd-ECM for 24 or 48 hours and the expression of several genes was evaluated using Western blot and RT-qPCR analysis (Supplemental Figure S3A). The results show that proteins such as PCNA, cyclin D1, Bcl-2, MMP2, MMP9, and Bcl-xL, expressed in abundance in cancer cells, were significantly downregulated in WHCO1 and MDA MB 231 cells after treatment with MSC-CM, coculture with MSCs, or when grown on fd-ECM for 24 hrs (Figures 1(a)-1(b), densitometric analysis in Supplemental Figure S3 B-C). However, there was an increase in p53 and p21 protein levels when MSCs were cocultured with cancer cells. Culturing cancer cells on fd-ECM resulted in significant increase in p21 and not p53 protein levels (Figures 1(a)-1(b), densitometric analysis in Supplemental Figure S3 B-C). MMP-2 and MMP-9 gelatinolytic activities were also significantly decreased when WHCO1 and MDA MB 231 cells were cocultured with MSCs and also in the presence of fd-ECM (Figures 1(c)-1(d)). Culture of WHCO1 and MDA MB 231 cells for a longer period (48 hrs), as described above, produced similar results (Figures 2(a)–2(d), densitometric analysis in Supplemental Figure S3 D-E). RT-qPCR analysis after 24 hrs of culture substantiated the results obtained by Western blot analysis (Supplemental Figure S4 A–H). Experiments done in the presence of both WJ-MSCs and fd-ECM showed that WJ-MSCs and fd-ECM synergistically downregulate PCNA, MMP-2, and Bcl-2 protein levels whilst the increase in p21 protein levels is more pronounced (Figures 3(a)-3(b)). To illustrate the specificity of this effect, coculture of WHCO1 and MDA MB 231 cells with WHCO1 and MDA MB 231 cells, respectively, as illustrated in Figures 3(c)-3(d), did not have an effect on Bcl-2 immunofluorescence staining (Figures 3(c)-3(d)). Coculture of both WHCO1 and MDA MB 231 cells with WI38 fibroblasts resulted in increased Bcl-2 and MMP-2 protein levels (data not shown).

### 3.2. WJ-MSCs and fd-ECM Inhibit WHCO1 and MDA MB 231 Cell Proliferation* In Vitro*

**Figure 4:**
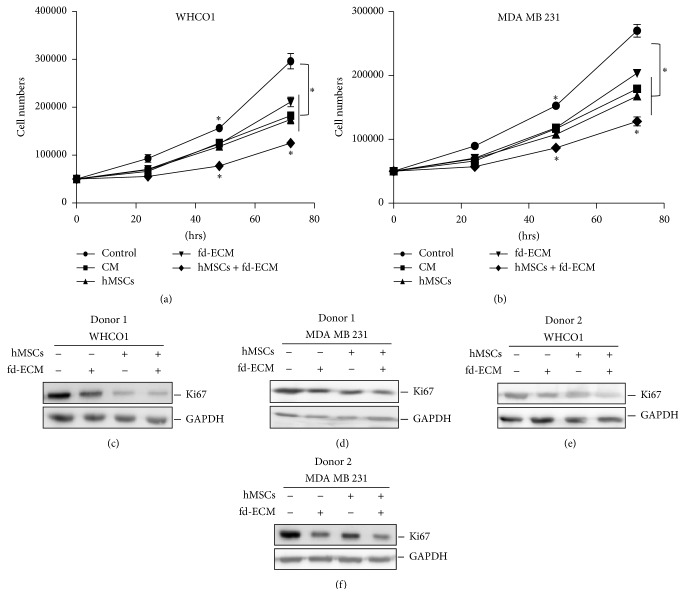
MSCs and the fd-ECM synergistically inhibit cancer cell proliferation. WHCO1 (a) and MDA MB 231 (b) cancer cells were cocultured with MSCs, cultured on fd-ECM, or in combination for the indicated time period. Control and treated cells were counted at the indicated times as described in Section 2. ((c)–(f)) Western blot analysis of cell lysates from control and treated WHCO1 and MDA MB 231 cells analyzed after 48 hrs of incubation using Ki67 antibody. Results obtained using MSCs from 2 donors are shown and are representative of all results obtained using WJ-MSCs from several other donors. Glyceraldehyde 3-phosphate dehydrogenase (GAPDH) was used as a loading control. Experiments were performed in triplicate and repeated 3 times.

To determine whether WJ-MSCs and the fd-ECM, alone or in combination, affect cancer cell proliferation, WHCO1 and MDA MB 231 cells were cocultured with MSCs and cultured on fd-ECM for 72 hrs. A semiquantitative assessment of whether the fd-ECM and coculturing of MSCs and cancer cells would affect cancer cell proliferation was done by counting the cells at 24 hr intervals and establishing growth curves. The merged results of growth curves show that MSCs and the fd-ECM reduced cancer cell proliferation compared to controls. WJ-MSCs together with fd-ECM reduced cancer cell proliferation more than individually (Figures 4(a)-4(b)). Western blot analysis of Ki67 in cancer cell lysates after 48 hrs of incubation also confirmed the decrease in cancer cell proliferation when MSCs from 2 donors were used (Figures 4(c)–4(f)). Coculturing WJ-MSCs and WHCO1 and MDA MB 231 cells whilst simultaneously plating the cancer cells on an fd-ECM resulted in a more pronounced reduction in Ki67 protein levels (Figures 4(c)–4(f)). This shows that the combined effect of WJ-MSCs and fd-ECM is synergistic. These results are representative of all data obtained using WJ-MSCs from different donors.

### 3.3. WJ-MSC- and Matrix-Mediated Downregulation of Gene Expression and Proliferation Occurs via a p21 Pathway

**Figure 5:**
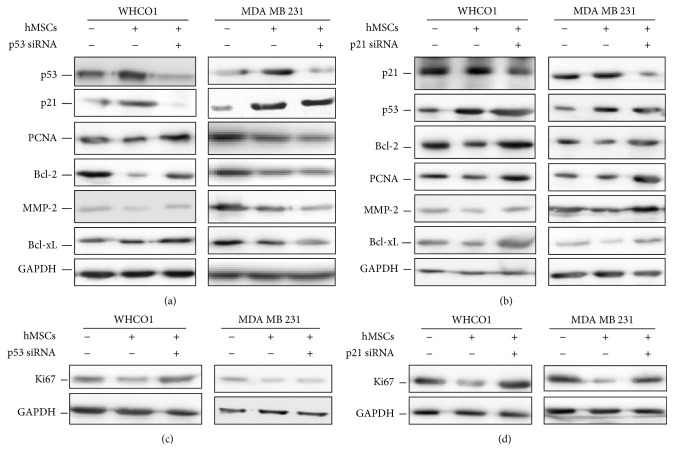
MSCs and matrix-mediated downregulation of WHCO1 and MDA MB 231 cellular gene expression occurs via a p21 pathway. (a) Western blot analysis of lysates from WHCO1 and MDA MB 231 cells cocultured with MSCs and transfected with p53 siRNA for 48 hrs showing expression of p53, p21, PCNA, Bcl-2, MMP-2, and Bcl-xL. (b) Western blot analysis of lysates from WHCO1 and MDA MB 231 cells cocultured with MSCs and transfected with p21 siRNA for 48 hrs showing expression of the same genes as in (a). (c) Western blot analysis of lysates from WHCO1 and MDA MB 231 cells cocultured with MSCs and transfected with p53 siRNA for 48 hrs showing expression of Ki67. (d) Western blot analysis of lysates from WHCO1 and MDA MB 231 cells cocultured with MSCs and transfected with p21 siRNA for 48 hrs showing expression of Ki67. Glyceraldehyde 3-phosphate dehydrogenase (GAPDH) was used as a loading control.

To confirm the involvement of both p53 and p21 in WJ-MSC-mediated gene downregulation, as observed in Section 3.1, WHCO1 and MDA MB 231 cells were transiently transfected with both p53 siRNA and p21 siRNA and cocultured with WJ-MSCs for 48 hours. Transfection with p53 siRNA resulted in a decrease in p21 protein levels and a reversal of the effect of coculture in WHCO1 cells (Figure 5(a); Supplemental Figure S5 A–D). However, suppression of p53 expression in MDA MB 231 cells did not decrease p21 protein levels and did not reverse the effect of coculture on several cancer cell genes downregulation (Figure 5(a)). p21 knockdown in WHCO1 and MDA MB 231 cells cocultured with WJ-MSCs did not affect p53 protein levels but was associated with a reversal of MSC-mediated cancer cell genes downregulation (Figure 5(b); Supplemental Figure S5 A–D). p21 knockdown was also associated with the reversal of fd-ECM-mediated downregulation of* PCNA* and* BCL-2* mRNA levels in both WHCO1 and MDA MB 231 cells. p21 knockdown also reversed the fd-ECM-mediated downregulation of Bcl-2 and MMP-2 protein levels (Supplemental Figure S5 E-F). Transfection with p53 and p21 siRNAs resulted in increased Ki67 protein levels cocultured WHCO1 cells (Figures 5(c)-5(d)). In MDA MB 231 cells, only p21 knockdown resulted in increased Ki67 protein levels (Figures 5(c)-5(d)). Together these results provide evidence that the cyclin-dependent kinase inhibitor p21 is induced by p53-dependent and p53-independent mechanisms in WHCO1 and MDA MB 231 cells, respectively, when cocultured with WJ-MSCs. p21 upregulation, in our coculture study and in the presence of fd-ECM, resulted in the downregulation of several cancer cell genes expression and is correlated with a reduced cancer cell proliferation.

### 3.4. WJ-MSC Coculture and fd-ECM Induce WHCO1 and MDA MB 231 Cancer Cell Apoptosis* In Vitro*

**Figure 6:**
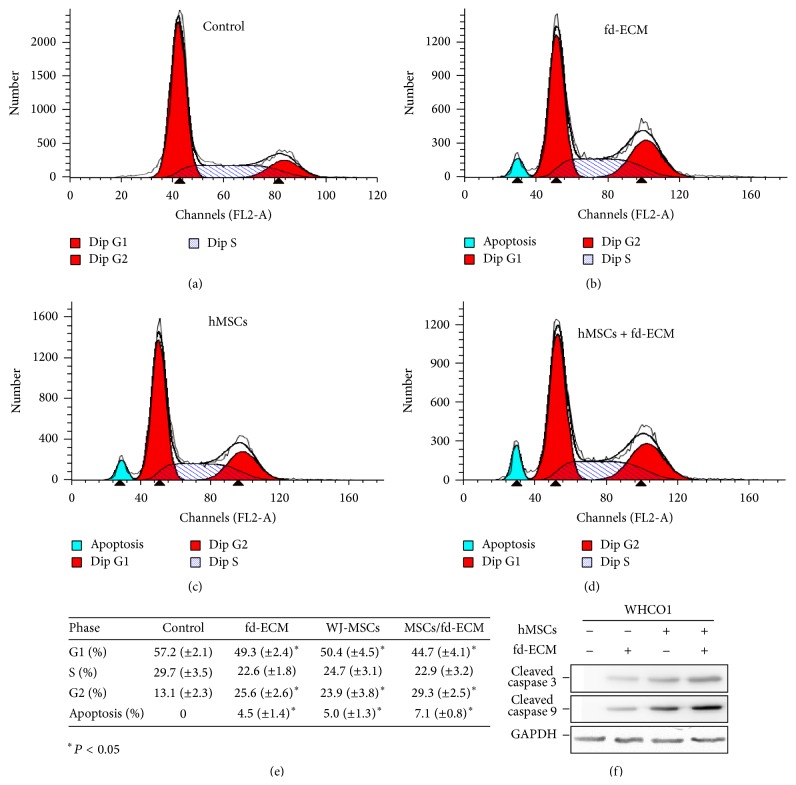
MSCs and fd-ECM synergistically induce cancer cell apoptosis* in vitro*. (a) Flow cytometric analysis of control WHCO1 cells showed no apparent apoptosis after 48 hrs of incubation. (b) WHCO1 cells were cultured on fd-ECM for 48 hrs and harvested and stained with propidium iodide for cell cycle analysis using flow cytometry. (c) WHCO1 cells were cocultured with MSCs for 48 hrs, harvested, and stained with propidium iodide for cell cycle analysis using flow cytometry. (d) Cocultured WHCO1 cells were plated on an fd-ECM for 48 hrs, harvested, and stained with propidium iodide for cell cycle analysis using flow cytometry. (e) Percentage of cells in each stage of the cell cycle after WHCO1 cells were cultured in MSC-CM, cocultured with MSCs, and cultured on fd-ECM. Four different experiments were pooled together. Data are presented as mean ± standard deviation. ^*∗*^ *P* < 0.05. (f) MSCs and fd-ECM synergistically induce cleaved caspases 3 and 9 expression in WHCO1 cells.

In order to investigate the effects of hMSCs coculture and the fd-ECM on cancer cell apoptosis, WHCO1 cells were analyzed after incubation for 48 hrs as indicated above by flow cytometry. Flow cytometric analysis showed that MSC-CM, coculture of WHCO1 cells with hMSCs, and plating WHCO1 on fd-ECM induced apoptosis in WHCO1 cells at 48 hours, with no obvious apoptosis in the control group (Figures 6(a)–6(d)). Coculture of WHCO1 cells with hMSCs resulted in 5.0% of the cells undergoing apoptotic cell death whilst plating WHCO1 cells on fd-ECM resulted in 4.5% of the cells undergoing apoptotic cell death (Figure 6(e)). Plating cocultured WHCO1 cells on fd-ECM resulted in 7.1% of the cells undergoing apoptotic cell death (Figures 6(d) and 6(e)). Western blot analysis showed increased caspase 3 and caspase 9 protein levels in the cocultured WHCO1 cells and also in the presence of the fd-ECM (Figure 6(f)). Plating cocultured WHCO1 cells on an fd-ECM resulted in increased cleaved caspases 3 and 9 expression (Figure 6(f)). A separate experiment showed that cocultured MDA MB 231 cells are induced to undergo apoptosis in the presence of WJ-MSC-CM and WJ-MSCs (Supplemental Figure S6 A–F). Thus, the effect of WJ-MSCs and fd-ECM is synergistic. p21 knockdown in WHCO1 cells cocultured with MSCs and plated on fd-ECM was associated with no apoptosis (Supplemental Figure S7 A–F). These results confirm the tumour suppressive effects of WJ-MSCs and the fd-ECM and implicate apoptotic pathways.

### 3.5. Both Coculture and fd-ECM Downregulate Tumorigenic Signaling Pathways

**Figure 7:**
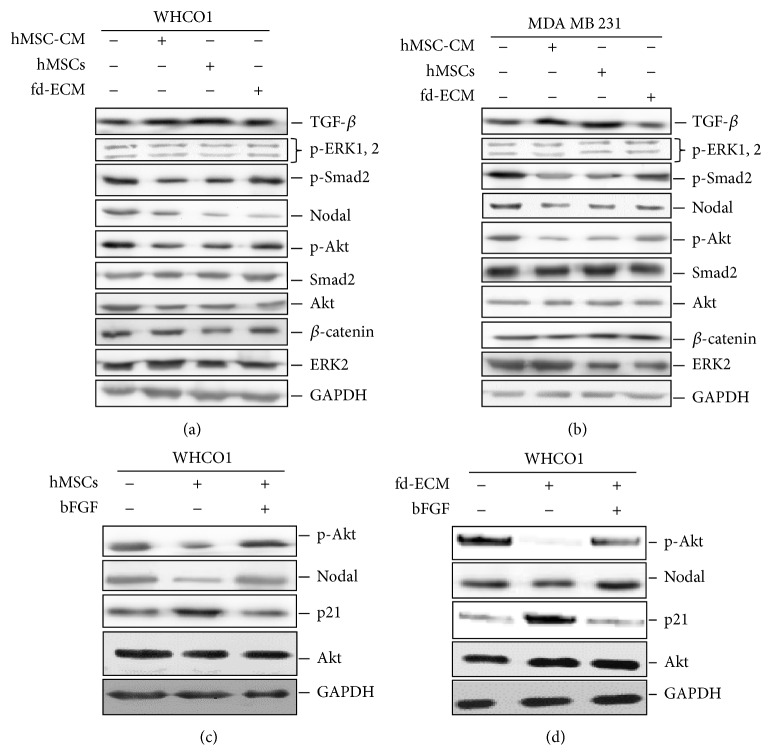
MSCs and the fd-ECM downregulate tumorigenic signaling pathways. (a) Western blot analysis of lysates from WHCO1 cells cocultured with MSCs or cultured on an fd-ECM for 48 hrs showing expression of TGF-*β*,* p*-ERK 1,2,* p*-Smad 2, Nodal,* p*-Akt, and *β*-catenin. (b) Western blot analysis of lysates from MDA MB 231 cells cocultured with MSCs or cultured on an fd-ECM for 48 hrs showing expression of the same genes as in (a). ((c)-(d)) Activation of the Akt pathway reverses the effect of hMSCs and fd-ECM on p21 expression. Cocultured WHC01 (a) and those plated on fd-ECM (b) were treated with 10 nM basic fibroblast growth factor for 48 hrs. Western blot analysis was performed on the protein lysates to determine p-Akt, Nodal, and p21 expression.

Many signaling pathways have been implicated in tumorigenesis [40]. We evaluated the expression of proteins involved in several signaling pathways implicated in tumorigenesis such as the *β*-catenin, PI3K/Akt, TGF-*β*1/Smads, Nodal, and the MEK-ERK signaling pathways [12, 16, 18, 22, 40]. Nodal is known to be expressed in a dysregulated fashion in several cancer cells including breast cancer cells [22, 40]. Coculture of MDA MB 231 and WHCO1 cells with WJ-MSCs resulted in an upregulation of TGF-*β* protein levels and a downregulation of Nodal, p-Smad2, and p-Akt protein levels in the cancer cell lines (Figures 7(a)-7(b)). No changes were observed in the protein levels of p-ERK 1, 2 and *β*-catenin. Culturing MDA MB 231 and WHCO1 cells on fd-ECM resulted in the downregulation of Nodal and p-Smad2 protein levels with no obvious changes in TGF-*β* and p-Akt protein levels (Figures 7(a)-7(b)). We show that the Nodal pathway is downregulated when cancer cells are cocultured with MSCs and cultured on an fd-ECM. Though the protein levels of *β*-catenin decreased slightly in the presence of MSCs and the fd-ECM, it was not significant (Figures 7(a)-7(b)). Activation of the PI3K-Akt pathway with the basic fibroblast growth factor (bFGF, final concentration 10 nM) was able to abrogate the effect of hMSCs and fd-ECM on p21 expression (Figures 7(c)-7(d)). This confirmed that p21 induction is downstream of the Akt pathway. These results suggest that the mesenchymal microenvironment, which is comprised of cells such as MSCs and the fd-ECM, downregulates several tumorigenic signaling pathways and inhibits the proliferation of cancer cells through various processes and not just a single pathway. From our data one of the potential mechanisms involves the activation of the PI3K-Akt pathway upstream of p21. p21 upregulation results in G2 phase cell cycle arrest leading to the induction of apoptosis.

### 3.6. Effect of WJ-MSCs and fd-ECM on WHCO1 and MDA MB 231 Cell Gene Expression Is Short- and Long-Term, Respectively

**Figure 8:**
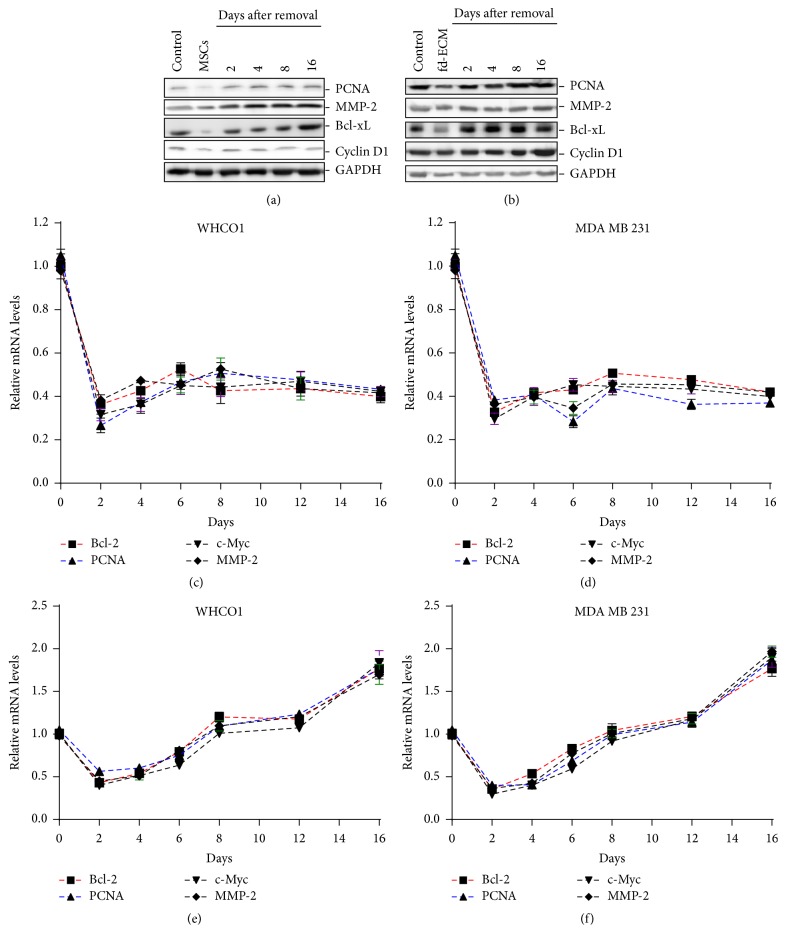
WHCO1 cells are able to recover from the effect of both MSCs and the fd-ECM. (a) Western blot analysis of WHCO1 lysates using antibodies against PCNA, MMP-2, Bcl-xL, and cyclin D1. WHCO1 cells were cocultured with MSCs followed by a 16-day recovery period. Recovery was achieved by removing WHCO1 cells from coculture and growing cells in normal DMEM media in plastic culture dishes. GAPDH was used as a loading control. (b) Western blot analysis of WHCO1 lysates using antibodies against PCNA, MMP-2, Bcl-xL, and cyclin D1. WHCO1 cells were cultured on fd-ECM for 48 hrs followed by a 16-day recovery period. Recovery was achieved by transferring and growing cells in plastic dishes. GAPDH was used as a loading control. ((c)-(d)) Prolonged culture of cancer cells on fd-ECM is able to maintain cancer cell gene downregulation. RT-qPCR analysis was performed to assess expression levels of PCNA, BCL-2, c-MYC, and MMP-2 in WHCO1 and MDA MB 231 cells cultured on fd-ECM over 16 days. ((e)-(f)) Prolonged coculture is unable to maintain WHCO1 and MDA MB 231 cellular gene downregulation. RT PCR analysis was performed to assess expression levels of PCNA, BCL-2, c-MYC, and MMP-2 in WHCO1 and MDA MB 231 cells cocultured with MSCs over 16 days.

WJ-MSCs- and fd-ECM-mediated downregulation of WHCO1 protein levels, as described above after coculture and plating on fd-ECM for 48 hrs, is reversible as WHCO1 cells revert back to producing normal levels of these proteins after several days of recovery (Figures 8(a)-8(b)). Recovery was achieved by transferring and growing cells in normal DMEM media and in plastic culture dishes. Longer incubation of WHCO1 cancer cells on the fd-ECM resulted in a sustained downregulation of* PCNA, BCL-2, MMP-2,* and* c-MYC* gene expression levels in WHCO1 and MDA MB 231 cells (Figures 8(c)-8(d)). However, longer incubation of WHCO1 and MDA MB 231 cells with WJ-MSCs did not result in sustained downregulation of* PCNA, BCL-2, MMP-2,* and* c-MYC* gene expression in the cancer cells indicating that the effect of coculture is transient. In fact, longer coculture of WHCO1 and WJ-MSCs up to 16 days resulted in a significant increase in expression of the above-mentioned genes compared to day 0 (control) (Figures 8(e)-8(f)). We therefore evaluated the effect of WHCO1 cells on WJ-MSCs gene expression over 24 days of coculture. The expression of *α*-SMA, the most reliable marker of tumour associated fibroblasts (TAFs) and vimentin, showed a clear and gradual increase in WJ-MSCs up to a maximum at day 16 when WJ-MSCs were cocultured with WHCO1 cells (data not shown). Our observations clearly show that over time MSCs exposed to esophageal and breast cancer cells differentiate and express markers of myofibroblast lineage.

## 4. Discussion

Mesenchymal stromal cells are pluripotent progenitor cells isolated from a variety of tissues including adipose tissue, placenta, Wharton's Jelly, and the bone marrow [1–4]. MSCs are known to give rise to many cells such as adipocytes, chondrocytes, and myocytes and are thus targeted in regenerative medicine. Beside their potential use in regenerative medicine MSCs are also targeted in cancer treatment. MSCs are known to be attracted to tumours* in vivo* where they contribute, positively or otherwise, to the development of the tumour stroma [4, 5, 41]. The literature is full of contradicting data on the effect of MSCs on tumour growth and several mechanisms have been invoked for the contrasting results such as vascular support, the presence of other cells in* in vivo* experiments, and the release of many chemokines [41–44]. One very important factor that might contribute to the contrasting results on the effect of MSCs on tumour growth is the variability of MSCs from different donors. A key aspect of reducing donor to donor MSC variability is the use of pooled MSCs in experiments. The use of pooled human MSCs is intended to overcome donor to donor variability and to provide a reliable and consistent source of MSCs for research. For short-term culture, pooling of MSCs would provide a consistent average population of cells which might not be true for long-term culture. It is important to note that the described experiments in this paper involved short-term culture of MSCs and were conducted using both single donor human MSCs and pooled MSCs (passages 5-6). Although previous studies have shown both tumour suppressive and oncogenic effects of MSCs, very little is known about the role of the tumour microenvironment in esophageal and breast cancer [41, 45, 46]. This is despite the fact that both oesophageal and breast cancers have a myofibroblastic tumour associated fibroblast-rich microenvironment [41]. Since the effect of MSCs on cancer cells, suppressive or oncogenic, depends on many factors such as the source of the MSCs and the type of cancer under study, we determined the effect of WJ-MSCs on WHCO1 and MDA MB 231 cancer cells. WJ-MSCs offer a good clinical alternative to adult MSCs in that they are easily obtained, have immune privileged status, lack stringent ethical concerns, and are not yet affected by the lifestyle of the individual [47, 48].

We have previously demonstrated that the fd-ECM reprograms fibroblasts and causes differentiation of human embryonic stem cells [32–34]. Thus, in this study we explored how the same fibroblastic microenvironment, represented by the fd-ECM, in addition to MSCs would affect human breast and esophageal cancer cells. Of significance, our study shows that both WJ-MSCs and the fd-ECM have tumour suppressive effects and this affects the expression of several WHCO1 and MDA MB 231 cancer cell genes, reprogramming the cancer cells to a less aggressive phenotype as shown by diminished proliferation and increased apoptosis. Our study shows that both WJ-MSCs and the fd-ECM inhibit WHCO1 and MDA MB 231 cancer cell proliferation via the p21 pathway. Induction of p21 induced G2 phase cell cycle arrest leading to apoptosis. We also found that the induced apoptosis was associated with the activation of caspases 3 and 9 indicating that both WJ-MSCs and the fd-ECM were affecting mitochondrial function. Given the ability of both WJ-MSCs and the fd-ECM to inhibit cancer cell growth and reduce expression of angiogenic growth factors (data not shown), both WJ-MSCs and the ECM would seem to be ideal candidates for new therapeutic strategies. Such strategies might take the form of the use of MSCs as seed cells for cancer treatment and the modification of organ ECM during chemotherapy or modification of the ECM of usual metastatic sites to prevent successful metastatic colonisation. If the utility of WJ-MSCs is confirmed, there is need for the cells to be administered to the tumour or human body for them to contribute to tumour therapy. The data from this study is very useful since the success of most cancer drugs is limited, hence requiring new approaches to improve the response to conventional therapy. Our data however calls for caution on the use of MSCs in cancer treatment as the effect of WJ-MSCs appears to be short-term and MSCs can differentiate to TAFs.

Although most cancer-related research has focused on tumour cells, accumulating evidence shows that stromal cells within the tumour microenvironment such as fibroblasts, MSCs, and endothelial and immune cells contribute in different ways to the development and progression of tumours [30, 41, 49–54]. During the early stages of tumour growth and metastatic colonisation, the ECM is mainly synthesised by other cells such as fibroblasts. Fibroblasts are probably the most abundant stromal cells in most cancers and are therefore involved in the development and progression of many epithelial cancers [41, 53, 54]. In addition, growth of tumour cells and invasion causes local tissue damage and injury which subsequently activates repair mechanisms and therefore attracts MSCs [51, 55, 56]. Separate studies on the influence of MSCs and various 2D ECMs on cancer cells have advanced our understanding of the epigenetic influence associated with the microenvironments of tumour cells and shed new clues on their implications [57–60]. However, tumour microenvironments include many components, cells, and the ECM, present at the same time. It is not surprising that conflicting data has been reported on the effect of MSCs and different ECMs on cancer cells [17, 21, 22]. These conflicting data are mainly due to many studies focusing on one component of the tumour stroma at a time and that some studies are conducted* in vitro* whilst others are conducted* in vivo* [61, 62]. We show in this study that WJ-MSCs and fd-ECM have a synergistic effect on WHCO1 and MDA MB 231 cell proliferation and gene expression. This is the first time this has been shown and we believe this study contribute substantively to our understanding of tumour cells and stromal cells interactions.

Recent studies showed that WJ-MSCs express high levels of several tumour suppressor genes which explain their lack of* in vivo* teratomas induction [47, 48, 63]. This might be the reason why WJ-MSCs inhibit tumour growth under several experimental setups. Several growth factors and chemokines are known to be secreted by WJ-MSCs and these can inhibit tumour growth in* in vitro* and* in vivo* experiments [63–67]. We examined the effect of WJ-MSCs and the fd-ECM on Bcl family proteins in cancer cells. Bcl proteins are key regulators of mitochondrial membrane function. Its downregulation together with Bcl-xL, MMP-2, MMP-9, and cyclin D1 provides a pathway through which proliferation is inhibited and apoptosis is induced. It has been shown that human umbilical cord mesenchymal stromal cells promote carcinoma growth when coinjected with esophageal carcinoma cells in nude mice [68, 69]. Their results differ from those reported in this* in vitro* study. Reasons for the different results could include the use of nude mice in their* in vivo* study as immune deficient mice do not closely reflect the real conditions of human beings. Another explanation could be that cancer cells secrete many chemokines such as IL6 and IL8 and can stimulate migration in cancer cells; thus, the decreased* IL6* and* IL8* expression observed in this study (data not shown) suggest that it is associated with the decrease in cancer survival genes such as* BCL-2, BCL-XL, MMP-2, *and* MMP-9*. MMP-2 and MMP-9 are the two most important proteins associated with tumour cell invasion and metastasis [68, 69]. We observed in this study that both MMP-2 and MMP-9 protein levels and activity are downregulated in the presence of WJ-MSCs and fd-ECM, clearly showing that both MSCs and fd-ECM have an effect on “cancer survival” genes.

Classic 2D cell culture methods have been shown to be insufficient for predicting tumorigenicity [26, 51, 70] and the one solution is the use of 3D ECMs. Therefore, in this study the effects of MSCs and 3D fibroblast-derived ECM on cancer cells were evaluated, separately and together. This 3D fibroblast-derived ECM was used to analyze cancer cells behaviour to recapitulate their invasion of mesenchymal tissue. We show here that both MSCs and 3D fd-ECM inhibited cancer cell growth and proliferation and increased apoptosis, and the effects appeared to be more pronounced when both were present. This inhibitory effect is transient and when removed from coculture and from fd-ECM cancer cells revert back to their tumorigenic nature. Inhibition of cancer cell proliferation can be equated to the initial inhibitory behaviour of a mesenchymal ECM on invading and metastatic cancer cells. Many studies have shown that both embryonic and mesenchymal microenvironments can repress tumour formation when implanted with aggressive human melanoma cells and also induce behaviours associated with a more normal phenotype [14, 15, 26, 30, 51, 70–73]. A study by Serebriiskii and coworkers [26] demonstrated that a fibroblastic or mesenchymal microenvironment influences human tumour epithelial cells and illustrated the potential of a 3D fd-ECM as an* in vitro* drug screening platform. 

Despite differences in fold gene changes both WHCO1 and MDA MB 231 cancer cell lines underwent apoptosis after exposure to fd-ECM and coculturing with MSCs. Many signaling pathways have been shown to be overexpressed in tumour cells including embryological pathways such as the Wnt/*β*-catenin and Nodal pathways, probably due to the lack of a regulatory mechanism to keep these pathways in check [34, 74]. The inhibition of the Nodal pathway may therefore abrogate tumorigenesis. The mesenchymal microenvironment provides a previously unexplored therapeutic entity for the regulation of aberrantly expressed Nodal in esophageal and breast cancers. It was observed in this study that MSCs were able to inhibit cancer cell growth in an* in vitro* experiment via downregulation of both Nodal and Akt signaling. The downregulation of the Nodal and Akt pathways in cancer cells by both MSCs and the fd-ECM suggests a potential therapeutic target based on an anti-Nodal and anti-Akt treatment. PI3K-Akt signaling is frequently dysregulated in cancer and can determine cell growth, proliferation, migration, and invasion [74]. The downregulation of Akt phosphorylation when cancer cells are cocultured with MSCs has been shown before [66, 75, 76]. The results obtained in this study might lead to the development of novel cancer therapeutics based on targeting the microenvironment leading to a reversal of the dedifferentiated, plastic tumour cell phenotype. Indeed, a number of treatments that target the extracellular components of tumours are in development [77, 78]. Recent studies have also shown the importance of the extracellular space in determining drug effectiveness and cancer cell survival [16, 17, 26, 66, 68–70, 76]. Therefore, targeting or modifying the ECM together with the use of drugs seems to be a potentially useful therapeutic strategy for enhancing the efficacy of therapeutic agents. Though therapeutic strategies will continue to target tumour cells, this study points to new therapeutic targets within the tumour microenvironment that if targeted can reduce their growth and possible metastasis. Our study highlights the importance of the tumour stroma in esophageal and breast cancer development and progression.

## 5. Conclusion

**Figure 9:**
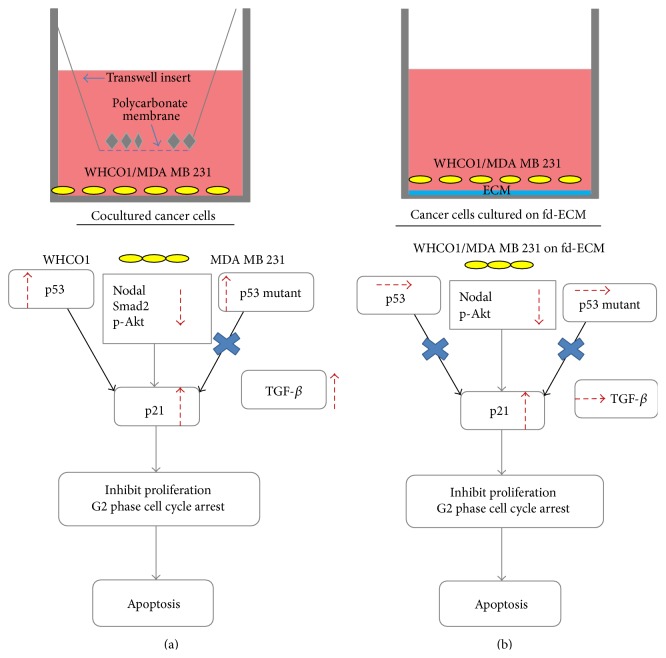
Summary of MSCs- and fd-ECM-mediated inhibition of cancer cell proliferation and G2 phase cell cycle arrest leading to apoptosis. (a) In the short term, the downregulation of tumorigenic pathways such as Akt and Nodal results in an upregulation of p21. p21 inhibits cancer cell proliferation and induces G2 phase cell cycle arrest leading to apoptosis. (b) fd-ECM-mediated inhibition of cancer cell proliferation and G2 phase cell cycle arrest occur via the same mechanism as for MSCs with the exception that there is no p53 and TGF-*β* upregulation.

This study showed that WJ-MSCs, in the short term, and, for the first time, the fd-ECM, downregulate tumorigenic signaling pathways such as PI3K-Akt and Nodal leading to p21 induction. p21 induction results in the inhibition of cancer cell proliferation and induction of G2 phase cell cycle arrest leading to apoptosis (Figures 9(a)-9(b)). A better understanding of the interplay involved between WJ-MSCs and cancer cells within the tumour microenvironment is needed for the development of better therapies. These findings warrant further research to understand the potential of MSCs and the fd-ECM as anticancer agents in breast and oesophageal cancer.

## Supplementary Material

Supplemental material include the phenotypic characterization of Wharton's Jelly derived MSCs. That involve the flow cytometric analysis of WJ-MSCs at passage 7 using antibodies to CD44-FITC, CD45-FITC, CD73-FITC, CD90-FITC and CD105-FITC. The lineage specific differentiation capacity of Wharton's jelly-derived MSCs was also assessed by evaluating the osteogenic, chondrogenic and adipogenic differentiation of the WJ-MSCs. A schematic representation of the experimental setup is also shown and the densitometric quantification of western blot gels, showing fold change in cancer gene expression compared to controls. Representative results showing the effect of MSCs and fd-ECM on cancer cell gene expression in 2 donors after 48 hrs of culture are also included plus the role of p21 in fd-ECM-mediated downregulation of cancer cell gene expression and how p21 knockdown prevents MSC-and fd-ECM-induced apoptosis in WHCO1 cells.
