# Supplementary material for: Wharton's Jelly-Derived Mesenchymal Stromal Cells and Fibroblast-Derived Extracellular Matrix Synergistically Activate Apoptosis in a p21-Dependent Mechanism in WHCO1 and MDA MB 231 Cancer Cells In Vitro
Source: Stem Cells Int. 2016 Jan 10;2016:4842134. doi: 10.1155/2016/4842134 (PMC4737007; doi:10.1155/2016/4842134)
Supplement: Supplementary file 1 — Supplemental material include the phenotypic characterization of Wharton's Jelly derived MSCs. That involve the flow cytometric analysis of WJ-MSCs at passage 7 using antibodies to CD44-FITC, CD45-FITC, CD73-FITC, CD90-FITC and CD105-FITC. The lineage specific differentiation capacity of Wharton's jelly-derived MSCs was also assessed by evaluating the osteogenic, chondrogenic and adipogenic differentiation of the WJ-MSCs. A schematic representation of the experimental setup is also shown and the densitometric quantification of western blot gels, showing fold change in cancer gene expression compared to controls. Representative results showing the effect of MSCs and fd-ECM on cancer cell gene expression in 2 donors after 48 hrs of culture are also included plus the role of p21 in fd-ECM-mediated downregulation of cancer cell gene expression and how p21 knockdown prevents MSC-and fd-ECM-induced apoptosis in WHCO1 cells. [file 4842134.f1.pdf]

Supplemental Table S1 Oligonucleotide primer sequences used for RT-qPCR

Gene	Forward	Reverse
GAPDH	5-GCTCTCCAGAACATCATCC-3’	5’-GCCTGCTTCACCACCTTC-3’
c-Myc	5’- AAGACTCCAGCGCCTTCTCTC -3’	5’- GTTTTCCAACTCCGGGATCTG -3’
PCNA	5’-CCA TCC TCA AGA AGG TGT TGG-3’	5’-GTG TCC CATATC CGC AAT TTT AT-3’
Cyclin D1	5’-ACAAACAGATCATCCGCAAACAC-3’	5’-CTTGGACTCCTCGGGGTTGT-3’
Bcl-2	5’-CTGCACCTGACGCCCTTCACC-3’	5’-CACATGACCCCACCGAACTCAAAGA-3’
Bcl-xL	5’-GATCCCCATGGCAGCAGTAAAGCAAG-3’	5’-CCCCATCCCGGAAGAGTTCATTCACT-3’
p21	5’- AGGGGACGGTCATCTACAACC -3’	5’- ATGGCCTTGCCATAGGCTGAG-3’
p53	5’-TTGGATCCATGTTTTGCCAACTGGCC -3’	5’- TTGAATTCAGGCTCCCCTTTCTTGCG -3’
MMP-2	5’-CCG CCT TTA ACT GGA GCA AA-3’	5’-TTT GGT TCT CCA GCT TCA GG-3’
MMP-9	5’- GAG ACA GCA TGG CCA AAT TA -3’	5’- CTC TAG AAA CTG CTG AGG GC-3'’
TGF-β	5’ GCAACAATTCCTGGCGATAC 3’	5’ AAGGCGAAAGCCCTCAAT 3’
α-SMA	5’AGCGTGGCTATTCCTTCGT 3’	5’ CCATCAGGCAACTCGTAACTC 3’
β-catenin	5-GCTCTGGAGTTCTCTCATCG-3	5-GGTGGAAGGACTTAGGTTTG-3
p16	5’- TTATTTGAGCTTTGGTTCTG-3’	5’- CCGGCTTTCGTAGTTTTCAT-3’
p21	5’-GAGGCCGGGATGAGTTGGGAGGA-3’	5’-CAGCCGGCGTTTGGAGTGGTAGAA-3’
CBFA1	5’-GGTTCCAGCAGGTAGCTGAG-3’	5’-AGACACCAAACTCCACAGCC-3’
OC	5’-GACTGTGACGAGTTGGCTGA-3’	5’-GGAAGAGGAAAGAAGGGTGC-3’
COL2	5’-CCTCTGCGACGACATAATCT-3’	5’-CTCCTTTCTGTCCCTTTGGT-3’
SOX9	5’-TAAAGGCAACTCGTACCCAA-3’	5’-ATTCTCCATCATCCTCCACG-3’
PPARG2	5’-ATGACAGCGACTTGGCAATA-3’	5’-GGCTTGTAGCAGGTTGTCTT-3’
LPL	5’-AGGAGCATTACCCAGTGTCC-3’	5’-GGCTGTATCCCGGGAGATGGA-3’

GAPDH: Glyceraldehyde 3-phosphate dehydrogenase; PCNA: proliferating cell nuclear antigen; Bcl-2: B-cell lymphoma 2; Bcl-xL: B-cell lymphoma-extra large; MMP 2: Matrix metallopeptidase 2; MMP 9: Matrix metallopeptidase 9; TGF-β: Transforming growth factor β; α-SMA: alpha-smooth muscle actin; CBFA1: Core-binding factor subunit alpha1; OC: Osteocalcin; COL2: Collagen type 2; SOX9: Sex determining region Y-box 9; PPARG2: Peroxisome proliferator-activated receptor gamma 2; LPL: Lipoprotein lipase.

Supplemental Figure Legends

Supplemental Figure S1 The phenotypic characterization of Wharton's Jelly derived MSCs (A-E) Flow cytometric analysis of WJ-MSCs at passage 7 using antibodies to CD44-FITC, CD45-FITC, CD73-FITC, CD90-FITC and CD105-FITC (F) The average percentage of cells staining positive for MSC surface epitopes as described above. The classical MSC phenotype is when the cells have the phenotype CD44+CD73+CD90+CD105+CD45- in ≥ 95% of the cell population. Different WJ-MSC preparation were used during the characterisation and for the sake of brevity we show representative results for 2 donors. Experiments were performed in triplicate and repeated at least 2 times.

Supplemental Fig S2 Lineage specific differentiation capacity of Wharton's jelly-derived MSCs (A-B) Osteogenesis was evaluated by analyzing mRNA levels of bone related markers such as *CBFA1* and *OC*. Control and induced samples were stained with alizarin red S. In osteogenic cultures (B) mineralization was visible (red stained calcium deposition) whereas this was absent in the control culture. Quantification of calcium deposition was done (C-D) Chondrogenesis was assessed by analyzing mRNA levels of cartilage related markers such as *COL2* and *SOX9*. Control and induced samples were stained with toluidine blue O. In chondrogenic cultures (D) staining of the proteoglycans (purple) was visible, whereas this was absent in control cultures. Quantification of glycosaminoglycan production is shown. (E-F) Adipogenesis was evaluated by analyzing relative mRNA levels of fat-related markers such as PPARG2 and LPL and quantification of lipid droplets is shown in (F).

Supplemental Fig S3 (A) Schematic representation of the experimental setup. Co-culture, plating of cells on fd-ECM and in combination. (B-E) Densitometric quantification of western blot gels, showing fold change in cancer gene expression compared to controls. (B) Fold change in WHCO1 gene expression after 24 hrs of co-culture and plating on fd-ECM (C) Fold change in MDA MB 231 gene expression after 24 hrs of co-culture and plating on fd-ECM (D) Fold change in WHCO1 gene expression after 48 hrs of co-culture and plating on fd-ECM (E) Fold change in MDA MB 231 gene expression after 48 hrs of co-culture and plating on fd-ECM. Data are presented as mean ± standard deviation. * $p < 0.05$.

Supplemental Fig S4 Representative results showing the effect of MSCs and fd-ECM on cancer cell gene expression in 2 donors after 48 hrs of culture. (A-B) RT-qPCR analysis was performed to assess expression of *PCNA* and *BCL-2* (A) and *P53* and *P21* (B) in control and treated WHCO1 cells after 48 hrs of culture. (C-D) RT-qPCR analysis was performed to

assess expression of *PCNA* and *BCL-2* (C) and *P53* and *P21* (D) in control and treated MDA MB 231 cells. (E-H) Donor 2 representative results (E-F) RT-qPCR analysis was performed to assess expression of *PCNA* and *BCL-2* (E) and *P53* and *P21* (F) in control and treated WHCO1 cells after 48 hrs of culture. (G-H) RT-qPCR analysis was performed to assess expression of *PCNA* and *BCL-2* (G) and *P53* and *P21* (H) in control and treated MDA MB 231 cells after 48 hrs of culture.

Supplemental Fig S5 The role of p21 in fd-ECM-mediated downregulation of cancer cell gene expression (A-B) WHCO1 and MDA MB 231 cells were plated on fd-ECM and transfected with p21 siRNA. RT-qPCR analysis was performed to assess expression of *PCNA* in control and treated WHCO1 and MDA MB 231 cells after 48 hrs of culture (C-D) WHCO1 and MDA MB 231 cells were plated on fd-ECM and transfected with p21 siRNA. RT-qPCR analysis was performed to assess expression of *BCL-2* in control and treated WHCO1 and MDA MB 231 cells after 48 hrs of culture. Data are presented as mean ± standard deviation.

* $p < 0.05$. (E-F) Western blot analysis of lysates from WHCO1 (E) and MDA MB 231 (F) cells plated on fd-ECM for 48hrs showing expression of Bcl-2 and MMP-2. Experiments were performed in triplicate and repeated at least 2 times.

Supplemental Fig S6 Fig 6 MSCs and fd-ECM induce cancer cell apoptosis *in vitro*. (A) Flow cytometric analysis of control MDA MB 231 cells showed no apparent apoptosis after 48 hrs of culture (B) MDA MB 231 cells were cultured in MSC conditioned media for 48 hrs and harvested, and stained with propidium iodide for cell cycle analysis using flow cytometry. (C) MDA MB 231 cells were co-cultured with MSCs for 48 hrs, harvested and stained with propidium iodide for cell cycle analysis using flow cytometry. (D) MDA MB 231 cells were cultured on an fd-ECM for 48 hrs, harvested and stained with propidium iodide for cell cycle analysis using flow cytometry. Experiments were performed in triplicate. (E) Summary showing percentage of cells in each stage of the cell cycle after MDA MB 231 cells were cultured in MSC-CM, co-cultured with MSCs and cultured on fd-ECM. Treated cells were compared to controls. This data is pooled from 4 different experiments using different MSCs preparations (F) Western blot analysis of lysates from MDA MB 231 cells grown in MSC-CM, co-cultured with MSCs or plated on fd-ECM for 48 hrs showing

expression of cleaved caspase 3 and 9. Experiments were performed in triplicate and repeated 3 times. * $p < 0.05$.

Supplemental Fig S7 p21 knockdown prevents MSC-and fd-ECM-induced apoptosis in WHCO1 cells (A) Flow cytometric analysis of control WHCO1 cells showed no apoptosis after 48 hrs of incubation (B-D) WHCO1 cells were cultured in MSC conditioned media (B), co-cultured with WJ-MSCs (C), cultured on fd-ECM (D) for 48 hrs and harvested, and stained with propidium iodide for cell cycle analysis using flow cytometry. (E) WHCO1 cells, co-cultured with MSCs, were transfected with p21 siRNA and cultured for 48 hrs. Cells were harvested and stained with propidium iodide for cell cycle analysis using flow cytometry (F) WHCO1 cells, plated on fd-ECM, were transfected with p21 siRNA and cultured for 48 hrs. Cells were harvested and stained with propidium iodide for cell cycle analysis using flow cytometry.

Supplemental Fig S1

A

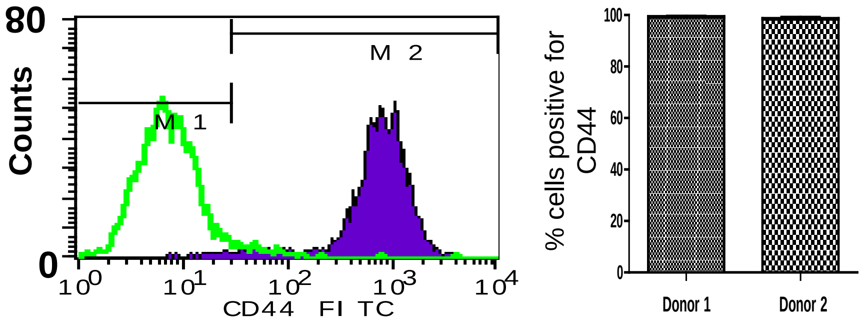

B

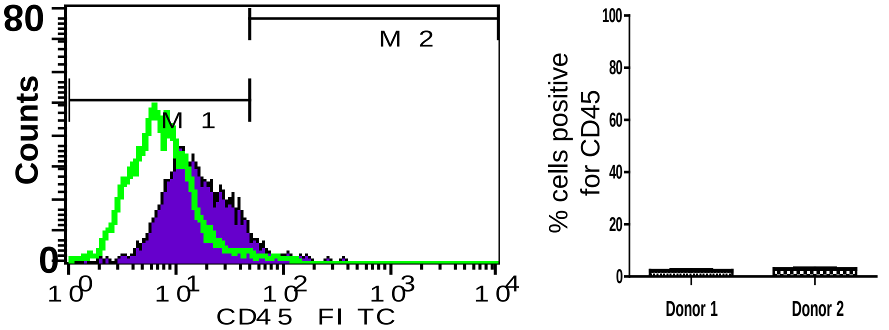

C

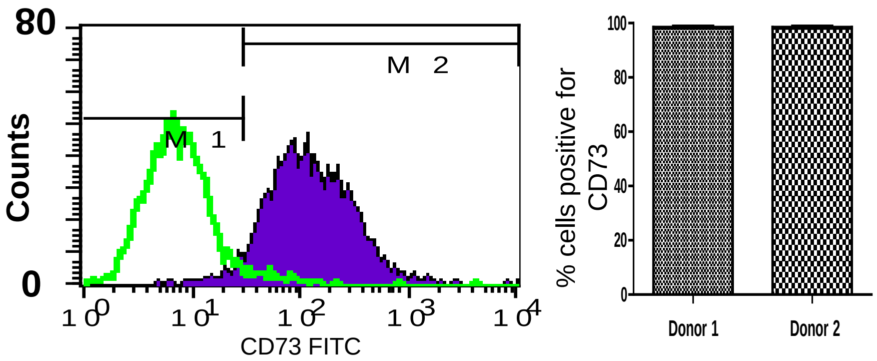

D

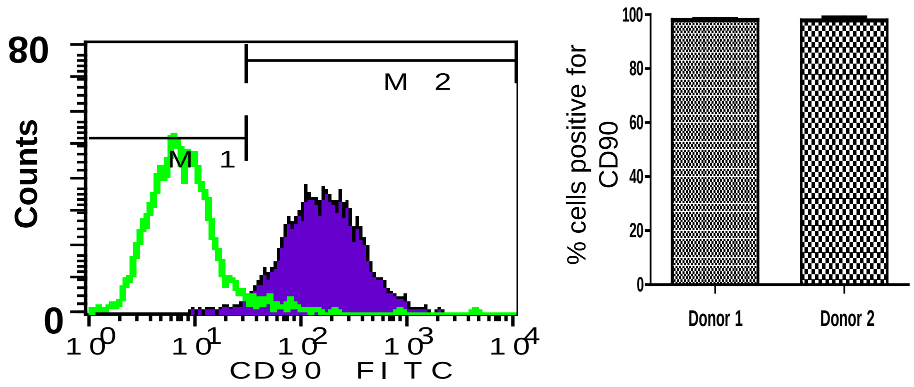

E

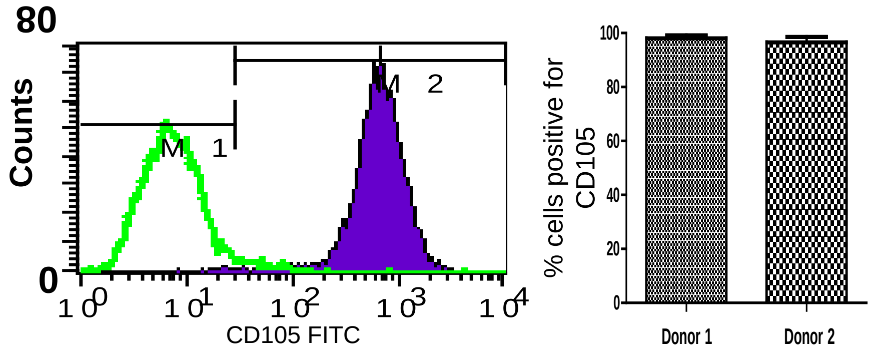

F

Marker	Mean % ± S.D	
	Donor 1	Donor 2
CD44	99.3 ± 0.20	98.5 ± 0.70
CD45	2.2 ± 0.60	2.9 ± 0.50
CD73	98.3 ± 0.70	98.3 ± 0.80
CD90	98.1 ± 0.50	97.9 ± 1.90
CD105	98.1 ± 1.90	96.6 ± 3.40

Supplemental Fig S2

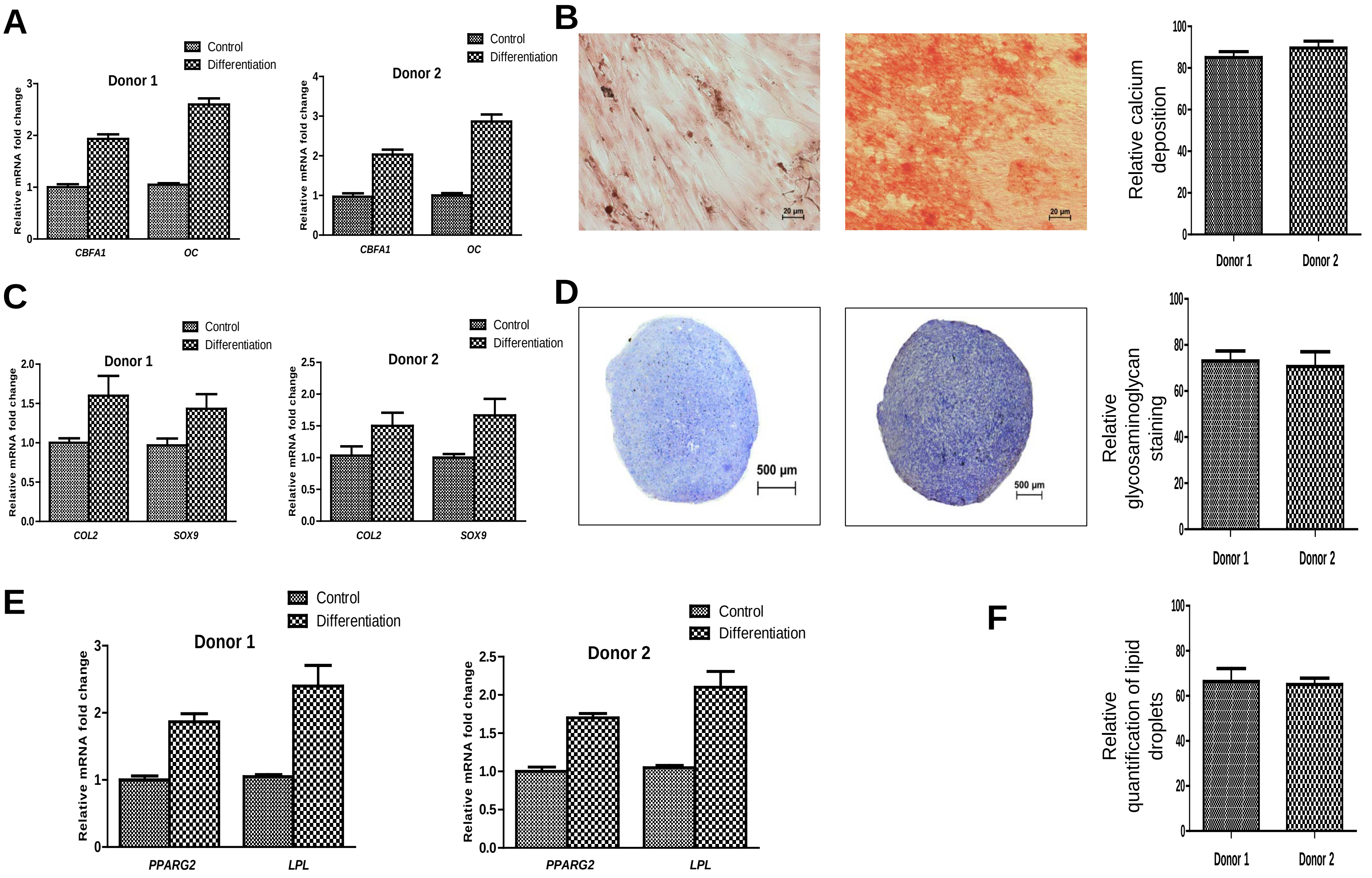

Supplemental Fig S3

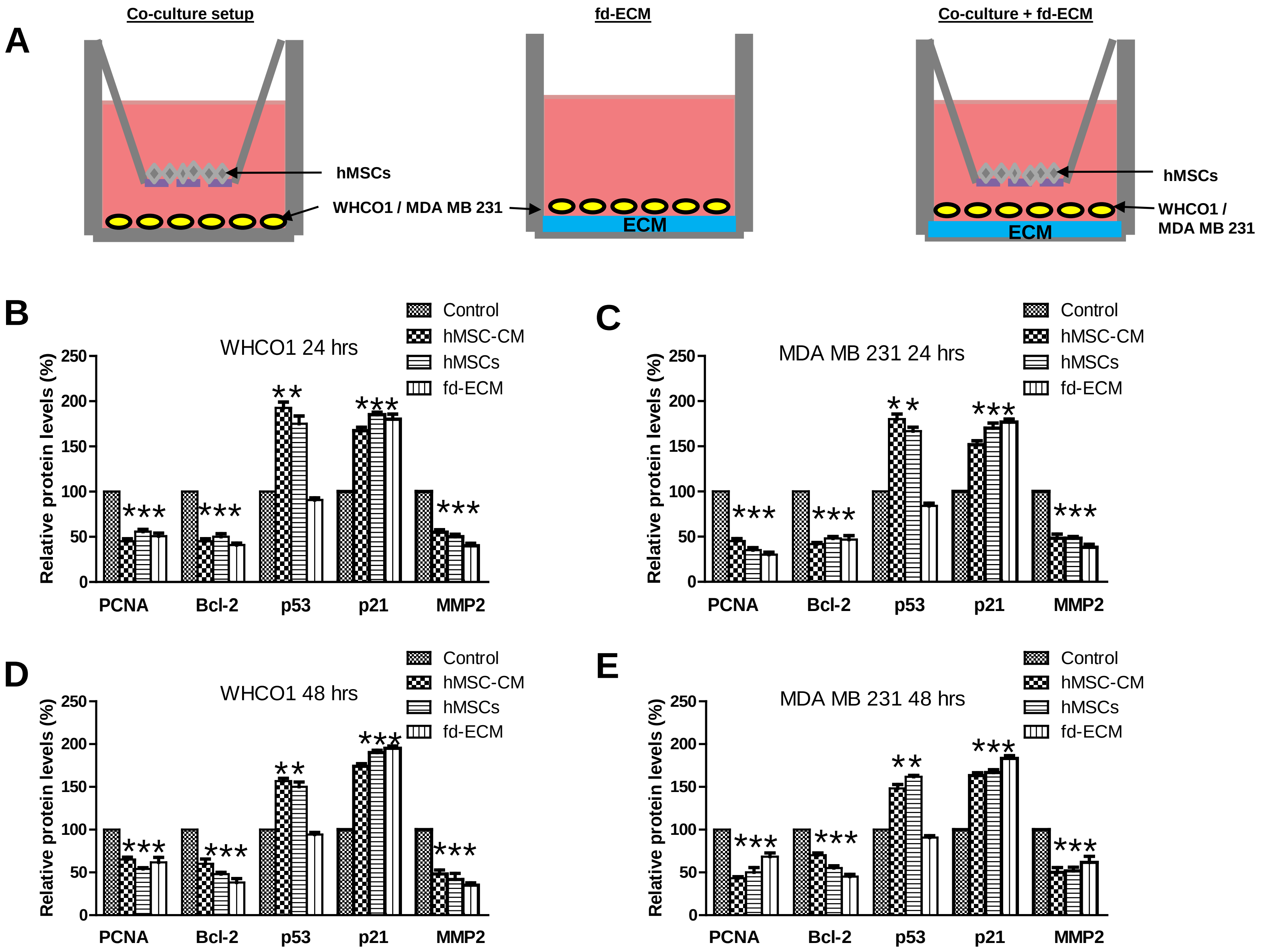

Supplemental Fig S4

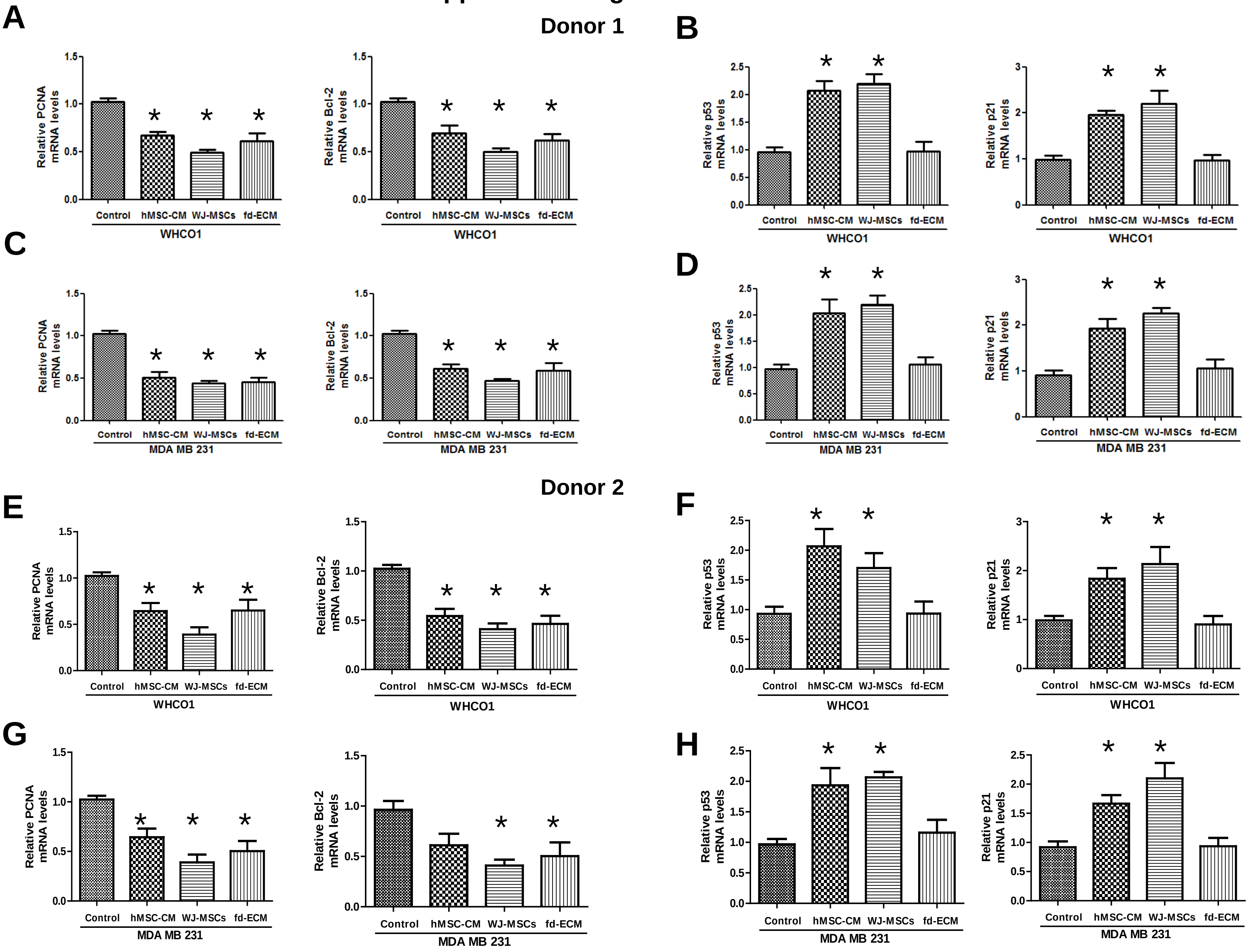

Supplemental Fig S5

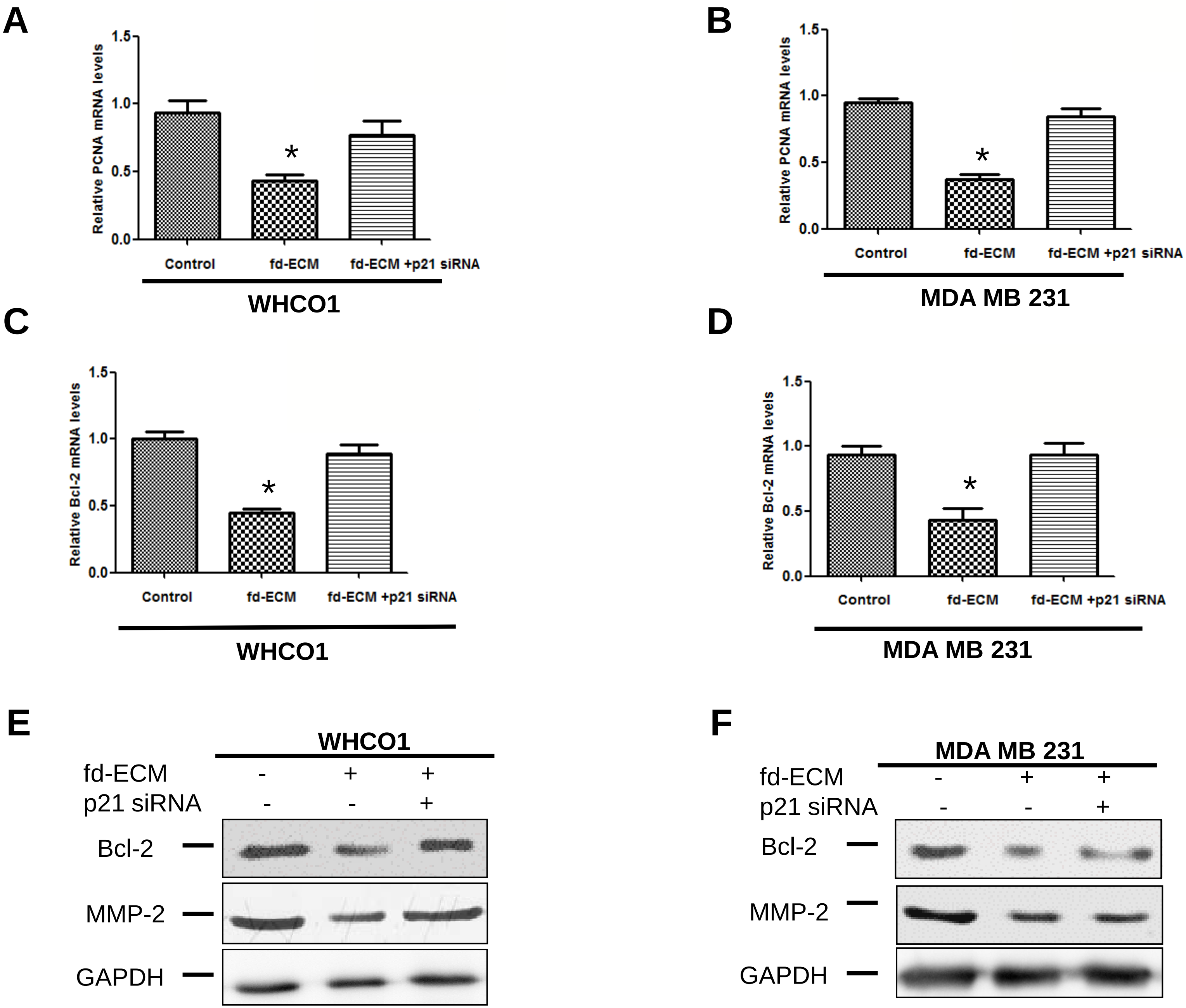

Supplemental Fig S6

A

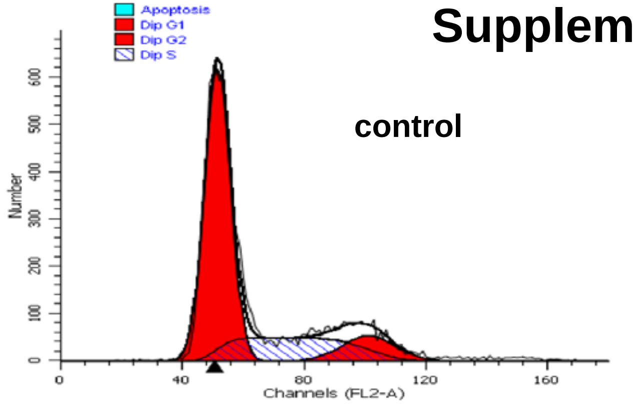

B

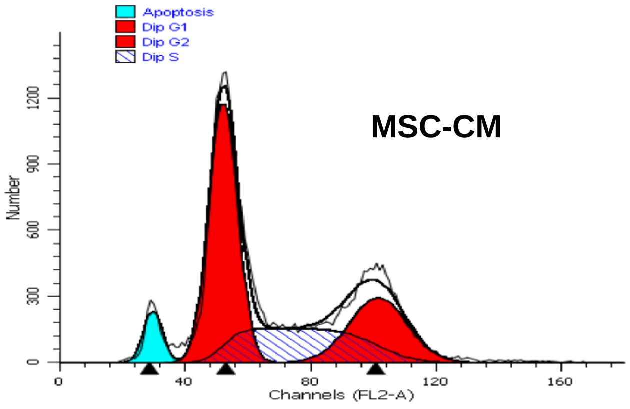

C

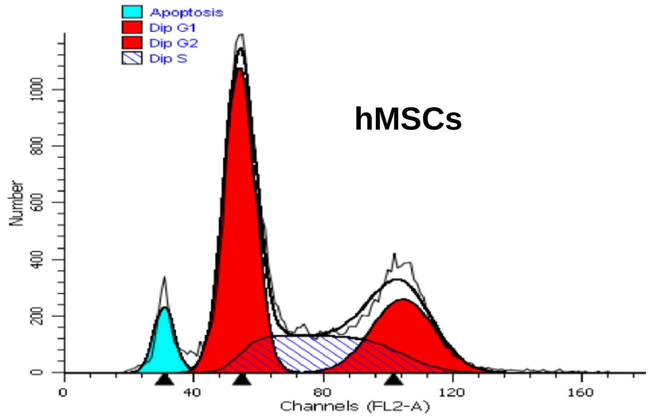

D

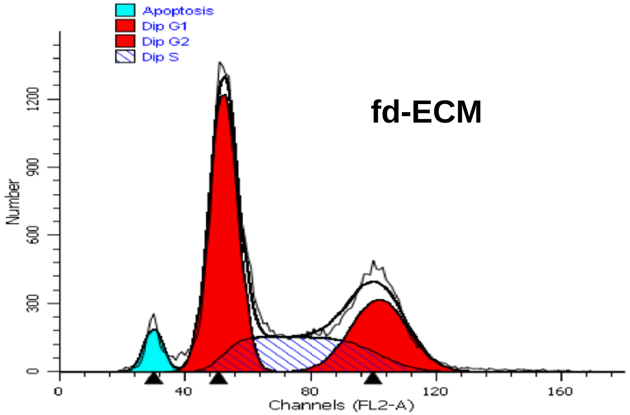

E

Phase:	Control:	CM:	WJ-MSCs:	fd-ECM:
% G1	64.9 (±2.3)	48.5 (±1.4)*	48.8 (±0.9)*	48.5 (±1.5)*
% S	24.1 (±1.2)	21.5 (±0.7)*	20.3 (±1.7)*	22.5 (±2.1)
% G2	11.0 (±0.5	27.1 (±1.1)*	26.2 (±0.7)*	26.8 (±3.1)*
% Apoptosis	0	5.9 (±0)*	6.7 (±1.1)*	4.8 (±0.7)*

* $p < 0.05$

F

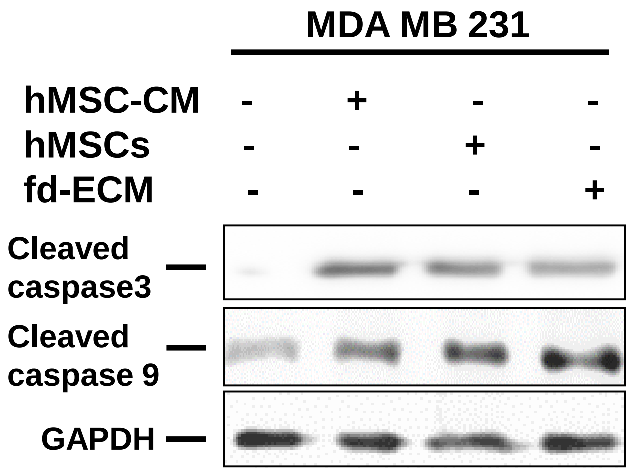

Supplemental Fig S7

A **control**

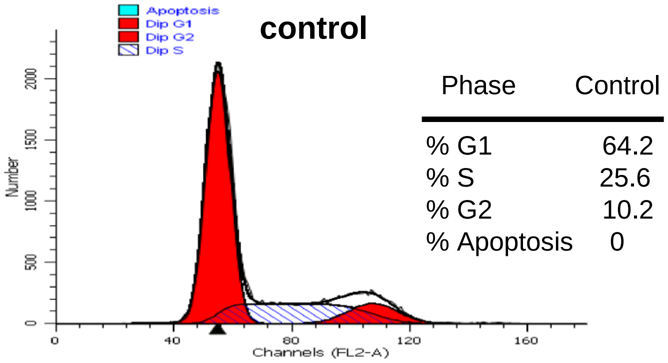

Phase	Control
% G1	64.2
% S	25.6
% G2	10.2
% Apoptosis	0

B **MSC-CM**

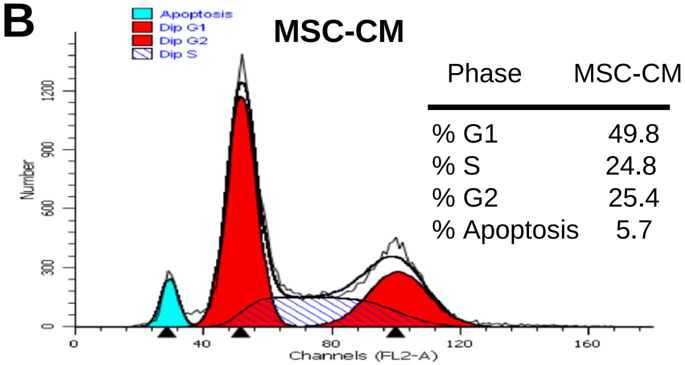

Phase	MSC-CM
% G1	49.8
% S	24.8
% G2	25.4
% Apoptosis	5.7

C **hMSCs**

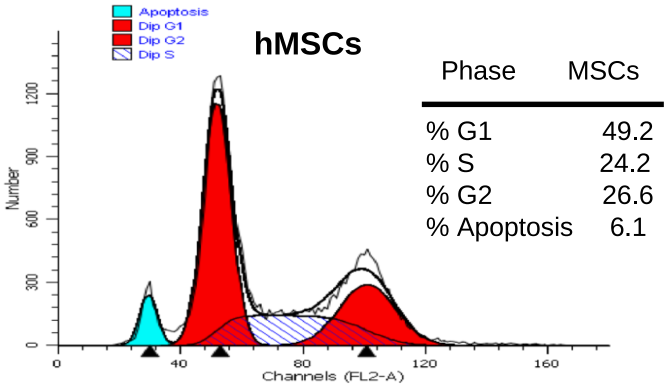

Phase	MSCs
% G1	49.2
% S	24.2
% G2	26.6
% Apoptosis	6.1

D **fd-ECM**

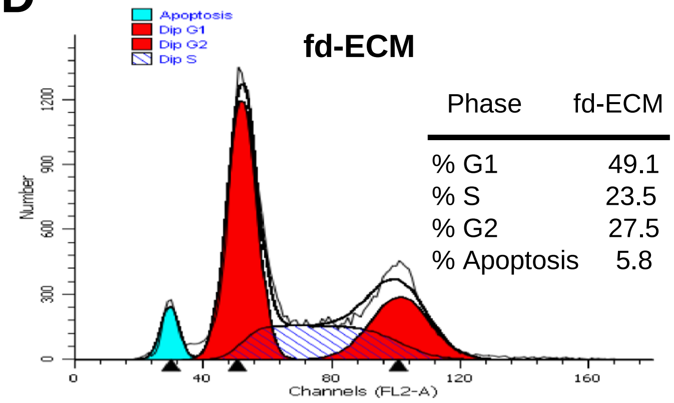

Phase	fd-ECM
% G1	49.1
% S	23.5
% G2	27.5
% Apoptosis	5.8

E **hMSCs + p21 siRNA**

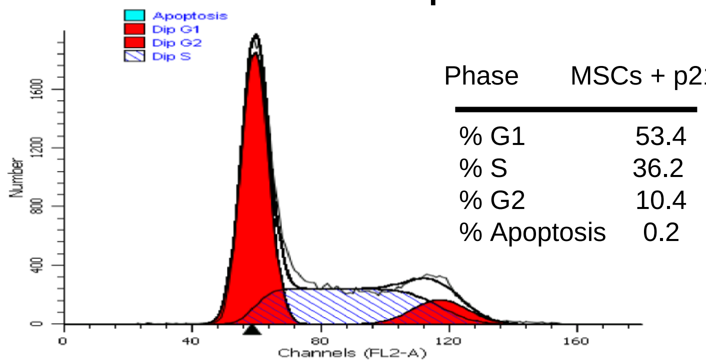

Phase	MSCs + p21 siRNA
% G1	53.4
% S	36.2
% G2	10.4
% Apoptosis	0.2

F **fd-ECM + p21 siRNA**

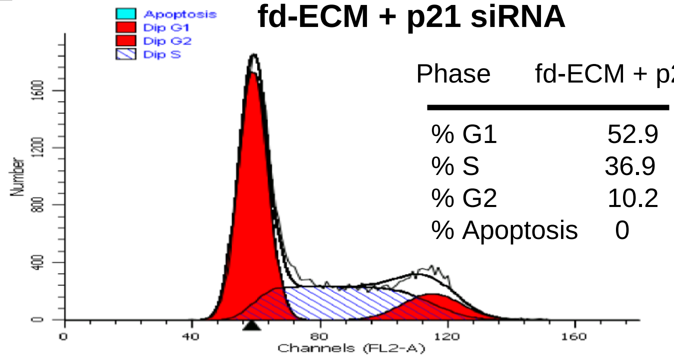

Phase	fd-ECM + p21 siRNA
% G1	52.9
% S	36.9
% G2	10.2
% Apoptosis	0
